# Rationale and design of the Renal Lifecycle trial assessing the effect of dapagliflozin on cardiorenal outcomes in severe chronic kidney disease

**DOI:** 10.1093/ndt/gfaf046

**Published:** 2025-03-07

**Authors:** Wisanne M Bakker, Hiddo J L Heerspink, Stefan P Berger, Christoph Wanner, Sunil V Badve, Clare Arnott, Alferso C Abrahams, Joost C van den Born, Tim C van Faassen, Carlo A J M Gaillard, Mariëlle A C J Gelens, Jose L Górris, Marc H Hemmelder, Lily Jakulj, Rob C M van Kruijsdijk, Dirk R J Kuypers, Peter van der Meer, Jeroen B van der Net, Heleen H Nijmeijer, Marc G Vervloet, Aiko P J de Vries, Michael Walsh, Angela Y Wang, Ron T Gansevoort, the Renal Lifecycle Trial Investigators, A Y Adema, A M Alphen, W A Bax, J Bayrak, H Boom, A H Boonstra, N Brinkman, E De Maar, Y De Waal, M Eshuis, M Hermans, D A Hesselink, E K Hoogeveen, J Huitema, W M T Jansen, J T Jonker, S W M Keet, S Konings, A Later, P Leurs, S Logtenberg, P Luik, G Ocak, A Ozyilmaz, J Rood, M Schouten, L Siddiqi-Nadery, C Siegert, J Slebe, F Stifft, T Van Bemmel, G F Van Breda, J Van der Heijden, J Van der Leeuw, A Van Eck van der Sluijs, R Van Etten, D Van Mil, F Waanders, J S Wiegersma, B Banas, K Budde, M Busch, M Girndt, M Guthoff, A L Herzog, B Hohenstein, M Schiffer, G Schlieper, M Schömig, B Schröppel, W Seeger, J Stegbauer, F Strutz, C Wanner, J Weinmann-Menke, M Zeier, J Holt, S Jesudason, K Keung, R Krishnasamy, H Kulkarni, A Makris, R Masterson, A Mather, D Palamuthusingam, E Pedagogos, B Smyth, V Srivastava, G Talaulikar, G Wong, M Wong, K Wyburn, T Dejagere, K Francois, W Lemahieu, E Mahieu, G Meeus, B S Alamo, M Blanco, J Buades, M L M Conde, J M Cruzado, N Garcia, M Goiciechea, F Gonzales, A B Porras, M Macia, M Marques, J J B Monzó, M A Munar, A Ortiz, M I S Saborido, M Salgueira, M J Soler, A Sancho, Y L A Liu, B W Teo, K Sreekanth, W Z I Lee

**Affiliations:** Department of Nephrology, University Medical Center Groningen, University of Groningen, Groningen, The Netherlands; Department of Clinical Pharmacy and Pharmacology, University Medical Center Groningen, University of Groningen, Groningen, The Netherlands; George Institute for Global Health, University of New South Wales, Sydney, NSW, Australia; Department of Internal Medicine, University Medical Center Groningen, University of Groningen, Groningen, The Netherlands; Department of Nephrology, University Hospital Wurzburg, Wurzburg, Germany; George Institute for Global Health, University of New South Wales, Sydney, NSW, Australia; George Institute for Global Health, University of New South Wales, Sydney, NSW, Australia; Department of Nephrology and Hypertension, University Medical Center Utrecht, Utrecht, The Netherlands; Department of Internal Medicine, University Medical Center Groningen, University of Groningen, Groningen, The Netherlands; Department of Clinical Pharmacy and Pharmacology, University Medical Center Groningen, University of Groningen, Groningen, The Netherlands; Department of Internal Medicine and Dermatology, University Medical Center Utrecht, Utrecht, The Netherlands; Division of Nephrology, Department Internal Medicine, Maastricht University Medical Center and Cardiovascular Research Institute CARIM, Maastricht, The Netherlands; Department of Nephrology, University Clinical Hospital, INCLIVA, Valencia, Spain; Division of Nephrology, Department Internal Medicine, Maastricht University Medical Center and Cardiovascular Research Institute CARIM, Maastricht, The Netherlands; Department of Nephrology, Erasmus University Medical Center, Rotterdam, The Netherlands; Division of Nephrology, Department Internal Medicine, Amsterdam University Medical Center and Dianet Dialysis Center, Amsterdam, The Netherlands; Department of Nephrology, Radboud University Medical Center, Nijmegen, The Netherlands; Department of Nephrology and Renal Transplantation, University Hospitals Leuven, Leuven, Belgium; Department of Cardiology, University Medical Center Groningen, Groningen, The Netherlands; Division of Nephrology, Department Internal Medicine, Albert Schweitzer Hospital, Dordrecht, The Netherlands; Department of Clinical Pharmacy and Pharmacology, University Medical Center Groningen, University of Groningen, Groningen, The Netherlands; Department of Nephrology, Radboud University Medical Center, Nijmegen, The Netherlands; Division of Nephrology, Department Internal Medicine, and Leiden Transplant Center, Leiden University Medical Center, Leiden University, Leiden, The Netherlands; Department of Medicine, McMaster University, Hamilton, ON, Canada; Department of Renal Medicine, Singapore General Hospital, Singapore; Department of Nephrology, University Medical Center Groningen, University of Groningen, Groningen, The Netherlands

**Keywords:** chronic kidney disease, dialysis, kidney transplantation, SGLT2 inhibitor, kidney failure

## Abstract

**Background:**

Several clinical trials have shown beneficial effects of sodium–glucose co-transporter 2 (SGLT2) inhibitors on kidney disease progression and cardiovascular morbidity and mortality in patients with chronic kidney disease (CKD) with and without type 2 diabetes mellitus. However, some subgroups of patients with CKD have been excluded from participation in these trials, such as patients with severely impaired kidney function, patients on dialysis and kidney transplant recipients.

**Methods:**

The Renal Lifecycle trial (NCT05374291) is a pragmatic, international, multicentre, investigator-initiated, randomized, placebo-controlled clinical trial planned to enrol ≈1500 patients with an estimated glomerular filtration rate (eGFR) ≤25 ml/min/1.73 m^2^, on haemodialysis or peritoneal dialysis or after a kidney transplant and an eGFR ≤45 ml/min/1.73 m^2^, who will be randomized 1:1 to receive either dapagliflozin 10 mg once daily or matching placebo.

**Results:**

The primary endpoint is a composite of heart failure hospitalization, all-cause mortality or, for those not on dialysis, kidney failure (start of dialysis >1 month, receiving a kidney transplant or death due to kidney failure). The trial is event driven, indicating that it will end after 468 first primary endpoint events have occurred, with a power of 80% and an α of 0.05 to detect a 25% relative risk reduction assuming an annual 12.5% incidence of the primary outcome. The secondary endpoints include a separate analysis of the incidence of each component of the primary endpoint in the overall trial population as well as the incidence of the combined primary endpoint in each of the three subgroups of patients. Other (exploratory) endpoints are efficacy, safety, tolerability, health-related quality of life and cognition.

**Conclusion:**

The Renal Lifecycle trial aims to investigate the effects of the SGLT2 inhibitor dapagliflozin compared with placebo on the incidence of kidney failure, heart failure, mortality and safety in three subgroups of patients with advanced CKD.

## Graphical Abstract

**Graphical Abstract:**
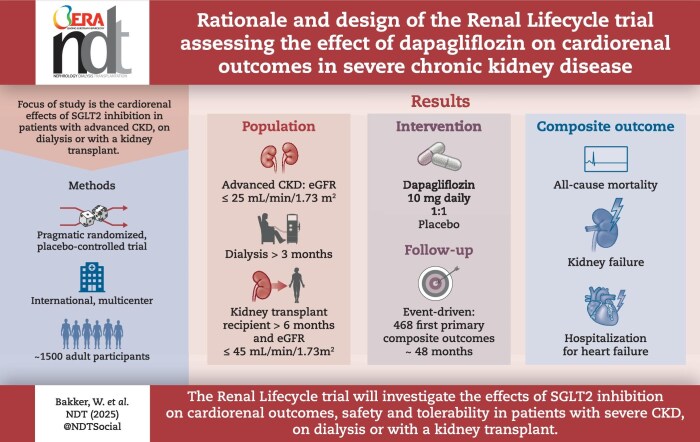

KEY LEARNING POINTS **What was known:**

- Cardiovascular outcome trials with sodium–glucose co-transporter 2 (SGLT2) inhibitors showed beneficial effects of these agents on heart failure and kidney outcomes.
- Secondary analyses from these cardiovascular outcome trials demonstrated that these benefits were consistent in patients with or without type 2 diabetes and with or without CKD.
- Dedicated randomized controlled trials performed specifically in patients with CKD showed similar beneficial effects on cardiorenal outcomes.

**This study adds:**

- Large subgroups of patients with CKD with the highest cardiovascular risk have been excluded from participation in the aforementioned trials, such as patients with severely impaired kidney function, patients on dialysis and kidney transplant recipients.
- The Renal Lifecycle trial will address this knowledge gap by investigating the cardiorenal protective effects, safety and tolerability of SGLT2 inhibition in these three subgroups with advanced CKD.

**Potential impact:**

- This trial may have a large clinical impact, as SGLT2 inhibition can potentially improve prognosis in patients with the highest risk for cardiorenal events.

## INTRODUCTION

Chronic kidney disease (CKD) has become one of the leading causes of death worldwide. The global prevalence of CKD is estimated to be 843.6 million people suffering from the early to late stages, and it is expected to increase due to an ageing population and an increasing prevalence of risk factors for CKD [1, 2]. The most common causes of CKD are hypertension and diabetes mellitus [3]. Therefore, many CKD treatment strategies focus on blood pressure management and/or diabetes regulation. Despite recent advancements in pharmacotherapy for the treatment of CKD, many patients still develop complications of CKD, indicating that there is a large unmet need for new therapeutic strategies.

Recently, a new class of medicines has been added to the therapeutic armamentarium to treat CKD, the sodium–glucose co-transporter 2 (SGLT2) inhibitors [4]. Cardiovascular outcome trials with these drugs showed beneficial effects of these agents on heart failure and kidney outcomes. Secondary analyses from these trials demonstrated that these benefits were consistent in patients with or without CKD [5]. Subsequently, three dedicated randomized controlled trials (RCTs) were performed in patients with CKD. First, the Canagliflozin and Renal Endpoints in Diabetes with Established Nephropathy Clinical Evaluation (CREDENCE) trial (NCT02065791) examined the effects of 100 mg canagliflozin compared with placebo in patients with type 2 diabetes mellitus (T2DM) and albuminuric kidney disease [estimated glomerular filtration rate (eGFR) 30–90 ml/min/1.73 m^2^ and a urine albumin:creatinine ratio (UACR) >300–≤5000 mg/g]. This trial was terminated early because an interim analysis showed a lower risk for both the primary renal composite endpoint {hazard ratio [HR] 0.70 [95% confidence interval (CI) 0.59–0.82]} and the secondary cardiovascular composite endpoint [HR 0.69 (95% CI 0.57–0.83)] [6]. Second, the Dapagliflozin and Prevention of Adverse outcomes in CKD (DAPA-CKD) trial (NCT03036150) investigated 10 mg dapagliflozin daily versus placebo in patients with an eGFR of 25–75 ml/min/1.73 m^2^ and a UACR ≥200 mg/g in patients with or without DM. A significantly lower risk for the primary outcome of incidence of halving of kidney function, end-stage kidney disease or all-cause mortality was found for dapagliflozin [HR 0.61 (95% CI 0.51–0.72)] [7]. Third, the EMPAgliflozin in patients with chronic KIDNEY disease (EMPA-KIDNEY) trial (NCT03594110) explored the effects of 10 mg empagliflozin versus placebo in patients with an eGFR of 20–45 ml/min/1.73 m^2^ or an eGFR of 45–90 ml/min/1.73 m^2^ with a UACR ≥200 mg/g with or without diabetes. A significantly lower risk was observed for the primary composite endpoint of progression of kidney disease or death due to cardiovascular causes [HR 0.72 (95% CI 0.64–0.82)] in both patients with and without diabetes [8]. These aforementioned results were obtained on top of standard care. Thus SGLT2 inhibitors are of added value in the treatment of patients with CKD, irrespective of the presence of diabetes.

Despite these promising results, large subgroups of patients with CKD with the highest cardiovascular risk profile have been excluded from participation in the aforementioned trials, such as patients with severely impaired kidney function (e.g. eGFR ≤30 ml/min/1.73 m^2^ in the CREDENCE trial, eGFR ≤25 ml/min/1.73 m^2^ in the DAPA-CKD trial and eGFR ≤20 ml/min/1.73 m^2^ in the EMPA-KIDNEY trial), patients on dialysis and kidney transplant recipients (KTRs). Recently, some data have been published that suggest the efficacy of SGLT2 inhibitors to improve prognosis in specifically these three subgroups of patients [7, 9, 10]. However, these preliminary data concern post hoc subgroup analyses [9] or non-randomized observational data [7, 10]. Thus there is persistent uncertainty whether SGLT2 inhibitors will be effective in patients with severely impaired kidney function, on dialysis or living with a kidney transplant. The Renal Lifecycle trial was designed to address this knowledge gap. It aims to investigate the efficacy and safety of dapagliflozin 10 mg once daily compared with placebo in these patients with advanced CKD.

## MATERIALS AND METHODS

### Trial design

The Renal Lifecycle trial is a pragmatic, international, multicentre, investigator-initiated, randomized, placebo-controlled, double-blind, superiority clinical trial enrolling patients in The Netherlands, Belgium, Germany, Spain, Singapore and Australia. In this trial, ≈1500 patients will be included with ≈500 patients per group (i.e. patients with advanced CKD, on dialysis or KTRs). The trial started in November 2022 with the initiation of the first site (UMC Groningen, Groningen, The Netherlands), and through December 2024 a total of 1125 participants have been randomized.

### Trial objectives

The primary endpoint is a composite of incident heart failure hospitalization, all-cause mortality or, for those not on dialysis, kidney failure (start of dialysis >1 month, receiving a kidney transplant or death due to kidney failure). The secondary outcomes consist of each component of the primary endpoint for the overall trial population and the combined primary endpoint in each of the three subgroups. Exploratory endpoints are time to new-onset T2DM (or post-transplant) in patients without diabetes, time to diuresis <200 ml/24 h in the dialysis subgroup (for those with residual diuresis), rate of change in eGFR over time in the patients with advanced CKD and KTRs, health-related quality of life (HRQoL) and cognitive functioning [measured by the Symbol Digit Modalities Test (SDMT)]. A detailed description of these outcomes is provided in Table 1. The trial is registered at clinicaltrials.gov with the identifier NCT05374291 and has EudraCT number 2023-508389-13-00. Details of the study can also be found on www.renal-lifecycle.com.

**Table 1:** Definition of endpoints.

Combined primary outcome
1. Kidney failure event A. Start of chronic dialysis (i.e. >1 month)^a^ B. Receiving a kidney transplant (not for the dialysis subgroup) C. Death due to kidney failure^b^ 2. Heart failure hospitalization: Heart failure hospitalization events will be assessed based on the following criteria (A–E). A confirmed event must meet all criteria (A–E). A. Admission to the hospital with a diagnosis of heart failure or prolongation of hospital stay due to heart failure. Heart failure must either be the reason for hospitalization or, if the hospitalization was for multiple concurrent reasons, must be likely to have required hospitalization if it had occurred alone or the other reasons would not have been sufficient to cause hospitalization if the HF had not occurred also. B. Specific treatment for heart failure, i.e. unplanned ultrafiltration/dialysis with volume removal, intravenous diuretics, vasoactive agents. C. Objective evidence of new or worsening heart failure by at least one of the following: • Peripheral oedema • Pulmonary crepitations/rales • Increased jugular venous pressure (JVP) • S3 gallop • Radiographic/imaging demonstrating pulmonary congestion (e.g. chest X-ray, computed tomography of the chest or lung ultrasound) • Increased NT-proBNP D. Symptoms (at least one of the following) • Dyspnoea • Decreased exercise tolerance E. Absence of an alternative explanation for the above. 3. All-cause mortality.
Secondary endpoints
• Time to kidney failure (in advanced CKD and transplant patients only) • Time to the first occurrence of heart failure hospitalization • Time to all-cause death
Exploratory endpoints
• Time to new-onset T2DM in patients without diabetes • Time to diuresis <200 ml/24 h in the dialysis subgroup^c^ • Rate of change in eGFR over time in patients with advanced CKD and KTRs • HRQoL measured by the EQ-5D and SF-12 • Cognitive functioning (measured by the SDMT)

a Both peritoneal dialysis and haemodialysis.

b Death due to kidney failure is defined as death due to kidney failure because dialysis treatment/a kidney transplant was deliberately withheld, not started or discontinued for any reason. Death due to kidney failure will be adjudicated by the Clinical Endpoint Adjudication Committee.

c Two consecutive values must both be <200 ml/24 h with the date of the first value counting as the time of occurrence of the event. In the CRF, it should be noted what the exact volume of diuresis was and whether it was collected over 24 h. If collection time deviates, the collection time (i.e. number of hours) should be recorded.

### Trial population

Patients >18 years of age can be included when they belong to one of three groups: patients with advanced CKD, defined as an eGFR ≤25 ml/min/1.73 m^2^; peritoneal dialysis or haemodialysis patients (≥3 months after start of dialysis); or KTRs with an eGFR ≤45 ml/min/1.73 m^2^ (≥6 months after transplantation). Only patients with advanced CKD have to be on a stable dose of angiotensin-converting enzyme (ACE) inhibitor or angiotensin receptor blocker (ARB) 4 weeks prior to the screening visit unless it is documented that the patient involved did not tolerate ACE inhibitors/ARBs. A detailed description of the inclusion and exclusion criteria is provided in Table 2. While the subject is in screening, tests for eligibility may be repeated once if there is historical evidence that the subject could qualify for the trial.

**Table 2:** Inclusion and exclusion criteria.

Inclusion criteria
In order to be eligible to participate in the randomized controlled double-blind trial the subject must meet the criteria for one of the three strata: • Patients with advanced CKD, i.e. an eGFR ≤25 ml/min/1.73 m^2^ • Haemodialysis and peritoneal dialysis patients (≥3 months after start of dialysis) • KTRs with an eGFR ≤45 ml/min/1.73 m^2^ (≥6 months after transplantation) • Age ≥18 years • Willing to sign informed consent • Predialysis patients with eGFR ≤25 ml/min/1.73 m^2^ have to be on a stable dose (no changes in dose or type of drug) of ACE inhibitors or ARBs for at least 4 weeks prior to the screening visit to be eligible to proceed to the randomization visit unless there is documented evidence that the patient does not tolerate an ACE inhibitor or ARB. These subjects will maintain their stable doses of ACE inhibitor or ARBs throughout the trial (when possible and tolerated by the patient). ACE inhibitors or ARBs are not required for patients on maintenance dialysis or KTRs.
Exclusion criteria
• Mentally incapacitated subjects (i.e. not able to sign informed consent) • Diagnosis of type 1 diabetes mellitus • Concurrent treatment with SGLT2 inhibitor • History of two or more urinary tract/genital infections during the last 6 months • Life expectancy <6 months in the opinion of the treating physician • Scheduled start of dialysis within 3 months or kidney transplantation within 6 months • Patients treated for a renal indication during the last 6 months with a course of systemic immunosuppressive agents or intensification of treatment with systemic immunosuppressive agents, such as patients with a kidney transplant and acute rejection or patients with granulomatosis with polyangiitis and a recent flare • Active malignancy aside from treated squamous cell or basal cell carcinoma of the skin • History of severe hypersensitivity or known severe hepatic impairment (Child–Pugh class C) • History of severe non-compliance with medical regimens or unwillingness to comply with the trial protocol • Pregnancy or breastfeeding • Presence of other transplanted organs besides a kidney transplant • Severe lactose intolerance^a^

a The trial medication contains so little lactose that most people with lactose intolerance do not suffer from this.

### Trial periods and procedures

#### Enrolment

Patients who meet the criteria for entry are asked by their treating physician if they are interested in participating. If so, they are invited for a screening visit, where trained local researchers verify inclusion and exclusion criteria. In patients who meet the inclusion criteria and do not have an exclusion criterion, they are asked to sign an informed consent (Appendix 2) by the local researcher. After informed consent has been obtained, information on medical history, concomitant medication, demographics and vital signs is obtained and a physical examination is performed together with urine and blood collection. All procedures per visit are specified in Table 3.

**Table 3:** Trial procedures.

		Double-blind treatment						
Procedure	Screening	Baseline						Study close-out visit
Visit	1	2	3	4	5	6	7–12…	EoS or EET^e^
Month	−0.5	0	0.5	3	6	12	Every 6 months	
Time window	−48 days		±14 days	±14 days	±28 days	±28 days	±28 days	
Informed consent^a^	x							
Randomization		x						
Significant medical history	x							
Physical examination	x	x^h^	x^h^	x^h^	x^h^	x^h^	x^h^	x^h^
Serum or urine pregnancy test^b^	x	x^b^	x^b^	x^b^	x^b^	x^b^	x^b^	x^b^
Blood sampling^f^	x	x	x	x	x	x	x	x
Early morning void urine sample^g^	x	x		x	x	x	x	x
24-hour urine collection^d–g^		x^a^		x	x	x	x	x
Residual kidney function^d^		x			x	x	x	x
Kt/V per week^d^		x			x	x	x	x
Vital signs^j^	x	x	x	x	x	x	x	x
EQ-5D and SF-12 questionnaires		x			x	x	x^i^	x
Biobanking (plasma and urine)		x		x				x
Endpoint assessment^c^			x	x	x	x	x	x
Dispense study medication		x		x	x	x	x	
Drug accountability (pill count)			x	x	x	x	x	x
SAEs and AESIs		x	x	x	x	x	x	x
Review medications	x	x	x	x	x	x	x	x
Cardiac MRI^k^		x				x		
Cardiac echocardiography^l^		x			x	x^n^		
Body composition measurement^l,m^		x			x	x^n^		
Additional biobanking (blood, urine and peritoneal effluent)^l^		x			x	x^n^		
Peritoneal dialysis modality, average ultrafiltration 4 weeks prior to study visit and PET data^l,o^		x			x	x^n^	x^n^	x^n^
6-minute walking test^l,m^		x			x	x^n^		
Kansas City Cardiomyopathy Questionnaire^l^		x			x	x^n^		
SDMT		x			x	x	x^i^	x

a Informed consent is obtained before any study specific procedure is done.

b WOCBP must have a negative serum or urine pregnancy test result (minimum sensitivity 25 IU/l or equivalent units of HCG) at screening or if pregnancy is suspected.

c At each visit the study sites will collect information about primary, secondary and exploratory endpoints.

d 24-hour diuresis, residual kidney function and Kt/V only recorded in dialysis patients. The most recent Kt/V values for each visit will be recorded with a time window of ±6 months.

e EoS: end of study; EET: early end of treatment. EET follow-up visit to be scheduled 14 ± 3 days after discontinuation of study medication.

f Visit 1 (screening) and all other visits: sodium, potassium, creatinine and urea; visit 2 (baseline) and EoS/EET: sodium, potassium, creatinine, urea, Hb, HbA1c, cholesterol, HDL cholesterol, LDL cholesterol, calcium, phosphate and PTH.

g Assessment of sodium, creatinine and albumin or protein (whichever is available).

h Only on indication.

i EQ-5D and SF-12 questionnaires and SDMT to be completed once every year after visit 6 until EoS/EET.

j Vital signs: heart rate, blood pressure and body weight.

k Cardiac MRI substudy only. The MRI will be performed within a time window of ±4 weeks.

l Cardiac echocardiography substudy only. The echocardiography, body composition measurement, Kansas City Cardiomyopathy Questionnaire and 6-minute walking test will be performed within a time window of ±4 weeks.

m If available on-site.

n Only participants who still receive peritoneal dialysis treatment at that time point and participate in the cardiac echography substudy.

o Collection of the most recent PET data if performed during routine medical care.

p Results of 24-hour urine collection at baseline should be determined within 3 months before the baseline visit. The other 24-hour urine collections should be taken within the time window of the visit.

#### Randomization and stratification

**Figure 1:**
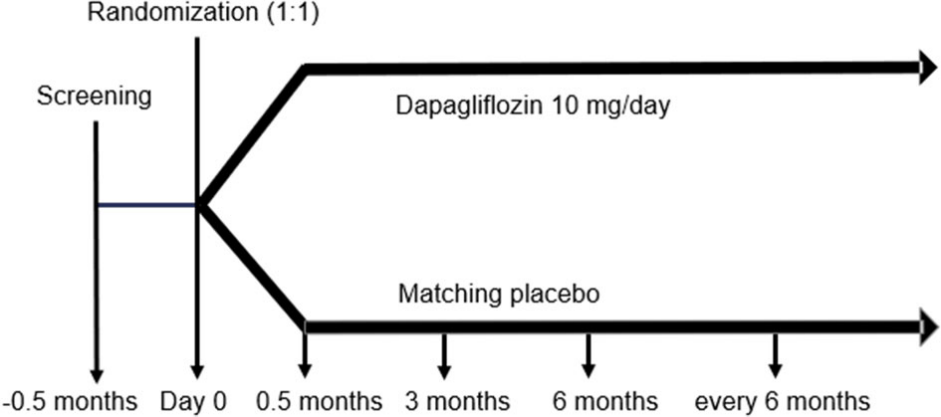
Overview of trial visits.

A randomization visit is scheduled ≈2 weeks after screening, where patients are randomized 1:1 to either dapagliflozin 10 mg once daily or matching placebo by the web-based randomization program ALEA using a minimization randomization method stratified based on subgroup (e.g. advanced CKD, dialysis, KTR), T2DM and centre. An overview of the trial visits is shown in Fig. 1. This is a double-blind trial, indicating that trial personnel, assessors, analysts and patients are blinded to the treatment until the end of the entire trial. Data are stored via electronic case report forms (eCRFs) in the web-based software program REDCap, with servers located at the UMC Groningen.

#### Follow-up trial visits

A safety visit is planned 2 weeks after randomization. Hereafter, in-person follow-up visits are planned at 3 months, 6 months and every 6 months thereafter. Adherence to the study drug is monitored by pill count. Assessment of HRQoL and cognitive function is done at baseline, 6 months, 12 months and once every year until the end of the trial. HRQoL is measured by the EuroQol 5-Dimensions (EQ-5D) and 12-item Short Form (SF-12) questionnaires and can be completed on paper or online based on the patient’s preference. Testing of cognitive function is done by the SDTM in an online app that patients can complete at home before the trial visit (Orikami, Nijmegen, The Netherlands) [11]. At the end of the trial, an end-of-trial visit will be scheduled.

In case patients opt to withdraw from using the study medication, an end-of-treatment visit is scheduled within 14 ± 3 days. After cessation of the study medication, patients are requested to continue follow-up visits to ensure the intention-to-treat principle. Temporary stopping of study medication is allowed in case of, for instance, a sudden decrease in eGFR ≥25% or other medical reasons, such as undergoing a surgical procedure, a scan with contrast or hospitalization. However, the temporary stop should not exceed 28 days.

#### Substudies

The Renal Lifecycle trial has several substudies. The cardiac magnetic resonance imaging (MRI) substudy will test the effects of dapagliflozin compared with placebo on indexed left ventricular mass at 12 months in a subset of 250 participants. The echocardiography substudy will compare the change in left ventricular global longitudinal strain from baseline to 6 months in a subset of 100 participants.

### Statistical considerations

#### Powering and sample size

The Renal Lifecycle trial is an endpoint-driven trial and will finish when 468 first primary trial outcomes have occurred. The sample size of ≈1500 patients is based on the primary trial outcome and provides 80% power to detect a 25% relative risk reduction assuming an annual 12.5% incidence of the composite primary outcome (α = 0.05). The definitive sample size can be adjusted according to the event rate observed during the trial. Based on previous trials with SGLT2 inhibitors in CKD, assumptions of an annual loss to follow-up of 1% and trial drug discontinuation of 5% are incorporated in the calculation. An 18-month recruitment period was predicted with a 30-month follow-up after the last patient inclusion, leading to a trial duration of ≈48 months, but this will be dependent on the rate of inclusion and the incidence rate of the primary outcome.

#### Primary outcome

Statistical analysis will be done based on the intention-to-treat principle. A time-to-event analysis will be performed and the Cox proportional hazards model will be applied for the composite primary endpoint to compare the treatment groups. In general, the analysis will use each patient’s last contact as the censoring date for patients without any primary events. The *P*-values, HRs and 95% CIs will be reported. Additionally, a calculation and plot of the cumulative incidence of the first occurrence of either the endpoint of kidney failure, heart failure hospitalization or all-cause mortality (composite primary endpoint) will be performed. The latter will be done for both the overall analysis and individual components.

#### Secondary outcome

A similar analysis as for the primary outcome will be performed. As the secondary trial endpoints comprise both an analysis of each component of the primary endpoint for the overall trial population and the combined primary endpoint for each subgroup, Cox proportional hazards models will be applied to investigate the treatment effects within each group separately. Patients who switch from dialysis to KTR will be considered to still be part of the dialysis group. The Fine–Gray model will be used to estimate cumulative incidence to take into account death as a competing risk for the kidney and heart failure endpoint. Event rates by treatment and HRs with 95% CIs will be reported and plotted for each subgroup. Moreover, treatment-modifying effects of baseline characteristics (e.g. age, sex, treatment modality, diabetes status) will be tested by interaction.

#### Safety and tolerability

Safety and tolerability will be assessed during the trial. Given the comprehensive experience with dapagliflozin, not all adverse events will be reported. However, serious adverse events (SAEs), adverse events (AEs) leading to (temporary) trial drug interruptions and adverse events of special interest (AESIs) [i.e. clinically significant hypoglycaemia, defined as glucose concentration <3.0 mmol/l (i.e. <54 mg/dl), ketoacidosis, urinary tract infections, genital infections] will be reported by number and percentage of patients. These AESIs will be investigated, as SGLT2 inhibitors have been suggested to be associated with these AEs. The investigator will report all SAEs to the sponsor within 24 hours after obtaining knowledge of the events. The Service Desk Clinical Research Office at UMC Groningen will act as the monitoring body and will be responsible for reporting SAEs and/or suspected unexpected serious adverse reactions (SUSARs) on behalf of the sponsor. The sponsor will inform the participating centres about SAEs and SUSARs and will take care of periodic safety reports for the central ethics committee and regulatory authorities (The Netherlands and Belgium). For the other sites, this will be performed by the local monitoring bodies according to the national reporting obligations. However, a low risk of AESIs is expected. Several placebo-controlled trials in patients with T2DM showed that the frequency of hypoglycaemia was similar in SGLT2 inhibitor treatment and placebo groups. Moreover, a meta-analysis of cardiovascular and CKD trials showed no increased risk for urinary tract infections and only a slightly increased risk for genital mycotic infections [12].

#### Ethical considerations

The trial has been approved in accordance with European medical ethical regulations in The Netherlands, Germany, Belgium and Spain and the national regulations for Australia. Medical ethical approval will be requested for the trial in Singapore. The trial is conducted in accordance with the International Conference of Harmonization Good Clinical Practice guidelines and follows the ethical principles stated in the Declaration of Helsinki.

#### Trial organization

A steering committee with members of university medical centres in The Netherlands, Belgium, Germany, Spain, Singapore and Australia has been appointed for a general overview of the trial and to provide advice on the design, protocol, conduct and data management. To ensure safety, an independent Data Safety Monitoring Board (DSMB) has been instituted to monitor the progress of the trial and the efficacy and safety of the intervention and to provide advice on continuation of the trial. Heart failure events are adjudicated by an independent clinical adjudication committee.

## DISCUSSION

SGLT2 inhibitors have been shown to have beneficial effects in patients with heart and kidney disease. A priori, it was hypothesized that SGLT2 inhibitors would be less efficient in patients with severely impaired kidney function, as these patients have a lower number of functioning nephrons and thereby less SGLT2 transporter to inhibit. Recently, a surprising finding was made in patients included in the DAPA-CKD trial who continued study medication after initiation of dialysis. In these patients, a lower mortality rate was observed in patients treated with dapagliflozin compared with placebo [7]. Of note, in this post hoc analysis, the number of patients who initiated dialysis was low (66 in the treatment group and 99 in the placebo group) and these patients were not randomized [13]. Consequently, these results should be interpreted with caution. This notwithstanding, this finding is remarkable. Moreover, Barreto *et al.* [14] showed that SGLT2 inhibitors were well tolerated in a small prospective study of 14 dialysis patients. An explanation for continued efficacy of SGLT2 inhibitors in patients with low kidney function was provided by a number of experimental studies suggesting that the beneficial effects of SGLT2 inhibitors on the heart and kidney could be caused by off-target effects other than renal SGLT2 inhibition alone. For instance, Juni *et al.* [15] showed in an experimental co-culture system of human cardiac microvascular endothelial cells and cardiomyocytes that empagliflozin had direct effects by restoring uraemic serum–induced systolic and diastolic dysfunction of cardiomyocytes. Moreover, Uthman *et al.* [16] showed that empagliflozin and dapagliflozin were able to block the effects of TNF-α in isolated endothelial cells of the human coronary vascular bed, leading to less production of reactive oxygen species and more production of the vasodilator substance nitric oxide. Recently, Billing *et al.* [17] performed a series of experimental studies in which they convincingly showed that SGLT2 inhibitors reduce the formation of uraemic toxins by changing the gut microbiome. In this way these drugs decrease the body’s exposure of uraemic toxins and the need for renal detoxification. This reflects yet another mechanism by which SGLT2 inhibitors could have beneficial effects in patients without kidney function. Therefore, based on these mechanisms, SGLT2 inhibitors seem to have potential in patients with advanced CKD and those treated with dialysis.

Currently, limited data are available on the efficacy and safety of SGLT2 inhibitors in KTRs. It is important to investigate this issue in this specific population to obtain a solid evidence-based rationale for treating (or not) these patients with SGLT2 inhibitors. Data obtained in CKD patients cannot automatically be extrapolated to KTRs, as mechanisms leading to disease progression may be different between these groups. However, preliminary data on SGLT2 inhibitors suggest efficacy in the KTR population. Lim *et al.* [10] showed in a retrospective study that KTRs with T2DM who had been treated with SGLT2 inhibitors had a better prognosis with respect to all-cause mortality, death-censored graft failure and doubling of serum creatinine compared with KTRs with T2DM not treated with SGLT2 inhibitors. In addition, no safety issues emerged in this study. Initially there was a concern that these drugs would cause an increased risk for urinary tract infections, which could pose a particular risk for KTRs, given the unphysiological anatomy of their urinary tract and the use of immunosuppressive agents. Therefore, the exclusion criterion of two or more urinary tract/genital infections in the last 6 months was implemented in our study protocol. However, the study by Lim *et al.* [10] found no increased risk for urinary tract infections and only a mildly increased risk for genital mycotic infections. This is in line with a meta-analysis that included safety data for most large-scale RCTs with SGLT2 inhibition in cardiovascular as well as kidney disease. This meta-analysis only showed an increased risk for genital infections, with a number needed to harm of 41, but not for urinary tract infections [12]. In general, genital infections usually can be easily treated by antimycotic agents and therefore safety does not seem to be compromised. Yet these safety data should also be interpreted with caution, being derived from non-KTR populations.

The aforementioned considerations were the rationale to design the Renal Lifecycle trial, which aims to assess the efficacy and safety of dapagliflozin 10 mg once daily compared with placebo in patients with an eGFR ≤25 ml/min/1.73 m^2^, dialysis patients and KTRs with an eGFR ≤45 ml/min/1.73 m^2^.

A unique aspect of this trial is that it is investigator initiated. Patents of SGLT2 inhibitors will run out in due time. Therefore, a large-scale, fully industry funded trial is no longer feasible. Fortunately, the present trial was made possible with funding by the Dutch Kidney Foundation, which received a large donation from the Piet Poortman Fonds [18]. During the trial, additional funding was obtained from the Australian government for participation of the Australian sites and from the pharmaceutical company AstraZeneca, which provided double-blind study medication and funded participation of the German sites. Nevertheless, funding is still limited and therefore the trial has been designed to be pragmatic. The frequency of clinic visits is therefore low and will not exceed the number of clinic visits needed for routine clinical care. Also, data collection for the trial will be done as much as possible as part of routine clinical care to minimize the burden for patients and investigators.

Some issues in our trial design merit in-depth description. The primary endpoint is the composite endpoint of kidney failure, heart failure hospitalization and all-cause mortality. This composite endpoint is used since previous trials showed a reduced risk with SGLT2 inhibitors for each of the three components [7, 9, 19, 6] and because each component is clinically important for patients. Of note, similar composite endpoints have been applied in other trials investigating SGLT2 inhibitors, such as the DAPA-CKD, CREDENCE, EMPA-KIDNEY and the EMPEROR-Preserved trials (NCT03057951) [7, 8, 6, 20]. In contrast to some other trials, hospitalization for heart failure was chosen as a cardiovascular outcome instead of major adverse cardiovascular events (MACE). It has been shown that treatment with the SGLT2 inhibitor dapagliflozin leads to a positive trend for heart failure but not MACE incidence [21]. Moreover, all-cause mortality was chosen (instead of cardiovascular death) since a prespecified analysis of the DAPA-CKD trial showed that the survival benefits of dapagliflozin were also seen for non-cardiovascular causes of death, which accounted for 41% of all deaths, whereas cardiovascular causes accounted for 37% of all deaths [13].

Based on the sample size calculation as described in the Methods section, ≈1500 patients have to be included with a trial duration of 4 years. It should be noted that the overall trial population consists of three subgroups, each consisting of ≈500 subjects, and consequently limited power to find significant results within each subgroup. Such a design including subgroups of patients has been applied before, for instance, in the Study of Heart and Renal Protection, in which patients with CKD and patients on dialysis (both haemodialysis and peritoneal dialysis) participated [22]. In the present trial, all three phases of a patient's renal lifecycle are combined (i.e. CKD, dialysis, kidney transplantation). Patients will continue participation if they reach another phase (e.g. advancing from CKD to dialysis or transplantation), hence the name of our trial. A temporary stopping of study medication may be applied in specific situations, such as a kidney transplant, but medication should be continued as soon as possible after the procedure. In case a patient starts dialysis treatment, study medication is also continued.

Specific entry criteria were formulated to define the three subgroups. Patients with advanced CKD should have an eGFR ≤25 ml/min/1.73 m^2^. Of note, only one RCT performed in CKD patients included subjects with an eGFR of 20–25 ml/min/1.73 m^2^. It was felt that the evidence for this eGFR subgroup is limited, allowing inclusion in the present study. Moreover, KTRs should have an eGFR ≤45 ml/min/1.73 m^2^. This was done to enrich for KTRs with a greater chance to reach an endpoint, as this chance is strongly dependent on baseline kidney function.

Patients with autosomal dominant polycystic kidney disease (ADPKD) were excluded from participation in prior SGLT2 outcome trials, as it is known that SGLT2 inhibitors increase vasopressin and that vasopressin receptor antagonists are renoprotective in this patient population [23]. In the Renal Lifecycle trial, patients with ADPKD are eligible, because the underlying cause of disease was deemed not to be of importance in patients on dialysis or in KTRs, while it was expected to have only limited relevance in patients with advanced CKD.

Another unique aspect of this trial is the high patient involvement, as patients provided input during the design of the trial. For instance, based on their advice, an early safety visit was replaced by a telephone consultation and the number of blood draws was minimized to decrease the burden for participants. During the execution of the trial, patients will be involved in a patient advisory committee that oversees and advises about patient inclusion, trial progress and implementation of the results of the trial.

No efficacy interim analysis is planned during the trial, as this is less imperative, because in general SGLT2 inhibitors have been found to be safe and well tolerated. This notwithstanding, safety will be monitored on an ongoing basis by the DSMB. In case of safety concerns, the trial can be terminated early.

In conclusion, previous trials that investigated the cardiorenal effects of SGLT2 inhibitors did not include patients with advanced CKD. Results of these trials can therefore not be directly extrapolated to these patients. The Renal Lifecycle trial aims to fill this knowledge gap, which may have a large clinical impact for this CKD population at highest risk. This trial will investigate the effects of the SGLT2 inhibitor dapagliflozin compared with placebo on the incidence of kidney failure, heart failure and mortality, as well as on safety, in patients with severely impaired kidney function, on dialysis or living with a kidney transplant.

## Supplementary Material

gfaf046_Supplemental_Files

## Data Availability

The data underlying this article will be shared upon reasonable request to the corresponding author.

## Appendix 1: APPENDIX 1: THE RENAL LIFECYCLE TRIAL COLLABORATORS

The Netherlands

A.Y. Adema (Medisch Centrum Leeuwarden, Leeuwarden), A.M. Alphen (Maasstad ziekenhuis, Rotterdam), W.A. Bax (Noordwest Ziekenhuisgroep, Alkmaar), J. Bayrak (Saxenburg Medisch Centrum, Saxenburg), H. Boom (Reinier de Graaf Gasthuis, Delft), A.H. Boonstra (Flevoziekenhuis, Almere), N. Brinkman (Medisch Spectrum Twente, Enschede), E. De Maar (Wilhelmina Ziekenhuis Assen, Assen), Y. De Waal (Ziekenhuis Bernhoven, Uden), M. Eshuis (Bravis Ziekenhuis, Roosendaal), M. Hermans (VieCuri Medisch Centrum, Venlo), D.A. Hesselink (Erasmus Medisch Centrum, Rotterdam), E.K. Hoogeveen (Jeroen Bosch Ziekenhuis, Den Bosch), J. Huitema (Laurentius Ziekenhuis, Roermond), W.M.T. Jansen (Martini Ziekenhuis, Groningen), J.T. Jonker (Alrijne Ziekenhuis Leiderdorp, Leiden), S.W.M. Keet (Maxima Medisch Centrum, Veldhoven), S. Konings (Catharina Ziekenhuis, Eindhoven), A. Later (Ziekenhuis St. Jansdal, Harderwijk), P. Leurs (Admiraal de Ruyter Ziekenhuis, Goes), S. Logtenberg (Diakonessenhuis, Utrecht; Dianet Utrecht, Utrecht), P. Luik (Meander Medisch Centrum, Amersfoort), G. Ocak (St. Antonius Ziekenhuis, Nieuwegein), A. Ozyilmaz (Dialyse Centrum Groningen, Groningen), J. Rood (Diapriva, Amsterdam), M. Schouten (Tergooi Medisch Centrum, Hilversum), L. Siddiqi-Nadery (Haaglanden Medisch Centrum, Den Haag), C. Siegert (OLVG, Amsterdam), J. Slebe (Elyse klinieken voor nierzorg, Kerkrade), F. Stifft (Zuyderland Medisch Centrum, Sittard), T. Van Bemmel (Gelre Ziekenhuis, Apeldoorn), G.F. Van Breda (Niercentrum aan de Amstel, Amstelveen), J. Van der Heijden (Spaarne Gasthuis, Hoofddorp/Haarlem), J. Van der Leeuw (Franciscus Gasthuis & Vlietland, Rotterdam), A. Van Eck van der Sluijs (Deventer Ziekenhuis, Deventer), R. Van Etten (Amphia Ziekenhuis, Breda), D. Van Mil (Universitair Medisch Centrum Groningen, Groningen), F. Waanders (Isala, Zwolle), J.S. Wiegersma (Ommelander ziekenhuis, Scheemda)

Germany

B. Banas (Universitaetsklinikum Regensburg, Regensburg), K. Budde (Charite Universitaetsmedizin Berlin, Berlin), M. Busch (Universitaetsklinikum Jena Klinik für Innere Medizin III, Jena), M. Girndt [Universitaetsklinikum Halle (Saale) Innere Medizin 2, Halle], M. Guthoff (Universitaetsklinikum Tuebingen, Tuebingen), A.L. Herzog (Universitaetsklinikum Wuerzburg, Wuerzburg), B. Hohenstein (Nephrologisches Zentrum Villingen- Schwenningen, Villingen-Schwenningen), M. Schiffer (Universitaetsklinikum Erlangen Medizin. Klinik 4–Nephrologie und Hypertensiologie, Erlangen), G. Schlieper (Zentrum für Nieren-, Hochdruck- und Stoffwechselerkrankungen, Hannover), M. Schömig (Dialysezentrum Heilbronn Ueberoertliche BAG für Nephrologie und Dialyse, Heilbronn), B. Schröppel (Universitaetsklinikum Ulm, Ulm), W. Seeger (Praxis für Dialyse und Nierenkrankheiten, Berlin), J. Stegbauer (Universitaetsklinikum Duesseldorf, Duesseldorf), F. Strutz (Nierenzentrum Wiesbaden, Wiesbaden), C. Wanner (University of Wuerzburg, Wuerzburg), J. Weinmann-Menke (Universitaetsmedizin Mainz, Mainz), M. Zeier (Universitaet Heidelberg, Heidelberg)

Australia

J. Holt (Wollongong Hospital, Wollongong), S. Jesudason (Royal Adelaide Hospital, Adelaide), K. Keung (Prince of Wales Hospital, Sydney), R. Krishnasamy (Sunshine Coast Hospital and Health Services, Birtinya), H. Kulkarni (East Metropolitan Health Service, Perth), A. Makris (Liverpool Hospital, Sydney), R. Masterson (Royal Melbourne Hospital, Melbourne), A. Mather (Royal North Shore Hospital, Sydney), D. Palamuthusingam (Royal Brisbane and Womens Hospital, Herston), E. Pedagogos [Western Health (Sunshine Hospital), St Albans], B. Smyth (St. George Hospital, Sydney), V. Srivastava (Townsville University Hospital, Townsville), G. Talaulikar (Canberra Health Services, Canberra), G. Wong (Westmead Hospital, Sydney), M. Wong (Concord Repatriation General Hospital, Sydney), K. Wyburn (Royal Prince Alfred Hospital, Sydney)

Belgium

T. Dejagere (Jessa Hospital, Hasselt), K. Francois (UZ Brussel, Brussel), E. Mahieu (AZ Glorieux, Ronse), G. Meeus (AZ Groeninge, Kortrijk)

For Spain

B.S. Alamo (Hospital Ramon y Cajal, Madrid), M. Blanco (Hospital CHUAC, A Coruna), J. Buades (Hospital de Son Llatzer, Palma de Mallorca), M.L.M. Conde (Hospital Arnau de Vilanova, Lleida), J.M. Cruzado (Hospital de Bellvitge, Barcelona), N. Garcia (Clinica Universitaria de Navarra, Navarra), M. Goiciechea (Hospital Gregorio Marañon, Madrid), F. Gonzales (Hospital Torrecardenas, Almeria), A.B. Porras (Hospital del Mar, Barcelona), M. Macia (Hospital de la Candelaria, Tenerife), M. Marques (Hospital Puerta de Hierro, Madrid), J.J.B. Monzó (Hospital Clinic, Barcelona), M.A. Munar (Hospital Son Espaes, Palma de Mallorca), A. Ortiz (Fundacion Jimenez Diaz, Madrid), M.I.S. Saborido (Hospital Germans Trias I Pujoil, Barcelona), M. Salgueira (Hospital Virgen Macarena, Sevilla), M.J. Soler (Hospital Vall d'Hebron, Barcelona), A. Sancho (Hospital Universitario Dr Peset, Valencia)

Singapore

Y.L.A. Liu (Khoo Teck Puat Hospital, Singapore), B.W. Teo (National University of Hospital System, Singapore), K. Sreekanth (Changi General Hospital, Singapore), W.Z.I. Lee (SengKang General Hospital, Singapore)

## Notes

A list of the Renal Lifecycle trial collaborators is provided in Appendix 1.
