# Supplementary material for: Rationale and design of the Renal Lifecycle trial assessing the effect of dapagliflozin on cardiorenal outcomes in severe chronic kidney disease
Source: Nephrol Dial Transplant. 2025 Mar 7;40(9):1746–55. doi: 10.1093/ndt/gfaf046 (PMC12394133; doi:10.1093/ndt/gfaf046)
Supplement: gfaf046_Supplemental_Files [file gfaf046_supplemental_files.zip › Supplementary -2 trial collaborators.docx]

**Appendix 2.** Renal Lifecycle trial collaborators

The Renal Lifecycle trial investigators are:

For the Netherlands

- Adema, AY, Medisch Centrum Leeuwarden, Leeuwarden
- Alphen, AM, Maasstad ziekenhuis, Rotterdam
- Bax, WA, Noordwest Ziekenhuisgroep, Alkmaar
- Bayrak, J, Saxenburg Medisch Centrum, Saxenburg
- Boom, H, Reinier de Graaf Gasthuis, Delft
- Boonstra, AH, Flevoziekenhuis, Almere
- Brinkman N, Medisch Spectrum Twente, Enschede
- De Maar, E, Wilhelmina Ziekenhuis Assen, Assen
- De Waal, Y, Ziekenhuis Bernhoven, Uden
- Eshuis M, Bravis Ziekenhuis, Roosendaal
- Hermans, M, VieCuri Medisch Centrum, Venlo
- Hesselink, DA, Erasmus Medisch Centrum, Rotterdam
- Hoogeveen, EK, Jeroen Bosch Ziekenhuis, Den Bosch
- Huitema, J, Laurentius Ziekenhuis, Roermond
- Jansen, WMT, Martini Ziekenhuis, Groningen
- Jonker, JT, Alrijne Ziekenhuis Leiderdorp, Leiden
- Keet, SWM, Maxima Medisch Centrum, Veldhoven
- Konings, S, Catharina Ziekenhuis, Eindhoven
- Later, A, Ziekenhuis St. Jansdal, Harderwijk
- Leurs, P, Admiraal de Ruyter Ziekenhuis, Goes
- Logtenberg S, Diakonessenhuis, Utrecht; Dianet Utrecht, Utrecht
- Luik, P, Meander Medisch Centrum, Amersfoort
- Ocak, G, St. Antonius Ziekenhuis, Nieuwegein
- Ozyilmaz, A, Dialyse Centrum Groningen, Groningen
- Rood, J, Diapriva, Amsterdam
- Schouten, M, Tergooi Medisch Centrum, Hilversum
- Siddiqi-Nadery, L, Haaglanden Medisch Centrum, Den Haag
- Siegert, C, OLVG, Amsterdam
- Slebe, J, Elyse klinieken voor nierzorg, Kerkrade
- Stifft, F, Zuyderland Medisch Centrum, Sittard
- Van Bemmel, T, Gelre Ziekenhuis, Apeldoorn
- Van Breda, GF, Niercentrum aan de Amstel, Amstelveen
- Van der Heijden, J, Spaarne Gasthuis, Hoofddorp/Haarlem
- Van der Leeuw, J, Franciscus Gasthuis & Vlietland, Rotterdam
- Van Eck van der Sluijs, A, Deventer Ziekenhuis, Deventer
- Van Etten, R, Amphia Ziekenhuis, Breda
- Van Mil, D, Universitair Medisch Centrum Groningen, Groningen
- Waanders, F, Isala, Zwolle
- Wiegersma, JS, Ommelander ziekenhuis, Scheemda

For Germany

- Banas, B, Universitaetsklinikum Regensburg, Regensburg
- Budde, K, Charite Universitaetsmedizin Berlin, Berlin
- Busch, M, Universitaetsklinikum Jena Klinik für Innere Medizin III, Jena
- Girndt, M, Universitaetsklinikum Halle (Saale) Innere Medizin 2, Halle
- Guthoff, M, Universitaetsklinikum Tuebingen, Tuebingen
- Herzog, AL, Universitaetsklinikum Wuerzburg, Wuerzburg
- Hohenstein, B, Nephrologisches Zentrum Villingen- Schwenningen, Villingen-Schwenningen
- Schiffer, M, Universitaetsklinikum Erlangen Medizin. Klinik 4 - Nephrologie und Hypertensiologie, Erlangen
- Schlieper, G, Zentrum für Nieren-, Hochdruck- und Stoffwechselerkrankungen, Hannover
- Schömig, M, Dialysezentrum Heilbronn Ueberoertliche BAG für Nephrologie und Dialyse, Heilbronn
- Schröppel, B, Universitaetsklinikum Ulm, Ulm
- Seeger, W, Praxis für Dialyse und Nierenkrankheiten, Berlin
- Stegbauer, J, Universitaetsklinikum Duesseldorf, Duesseldorf
- Strutz, F, Nierenzentrum Wiesbaden, Wiesbaden
- Wanner, C, University of Wuerzburg, Wuerzburg
- Weinmann-Menke, J, Universitaetsmedizin Mainz, Mainz
- Zeier, M, Universitaet Heidelberg, Heidelberg

For Australia

- Wong, M, Concord Repatriation General Hospital, Sydney
- Holt, J, Wollongong Hospital, Wollongong
- Jesudason, S, Royal Adelaide Hospital, Adelaide
- Krishnasamy, R, Sunshine Coast Hospital and Health services, Birtinya
- Kulkarni, H, East Metropolitan Health Service, Perth
- Keung, K, Prince of Wales Hospital, Sydney
- Makris, A, Liverpool Hospital, Sydney
- Masterson, R, Royal Melbourne Hospital, Melbourne
- Mather, A, Royal North Shore Hospital, Sydney
- Palamuthusingam, D, Royal Brisbane and Womens Hospital, Herston
- Pedagogos, E, Western Health (Sunshine Hospital), St Albans
- Smyth, B, St. George Hospital, Sydney
- Srivastava, V, Townsville University Hospital, Townsville
- Talaulikar, G, Canberra Health Services, Canberra
- Wong, G, Westmead Hospital, Sydney
- Wyburn, K, Royal Prince Alfred Hospital, Sydney

For Belgium

- Dejagere, T , Jessa hospital, Hasselt
- Francois, K, UZ Brussel, Brussel
- Lemahieu, W, Hospital Imelda, Bonheiden
- Mahieu, E, AZ Glorieux, Ronse
- Meeus, G, AZ Groeninge, Kortrijk

For Spain

- Alamo, BS, Hospital Ramon y Cajal, Madrid
- Blanco, M, Hospital CHUAC, A Coruna
- Buades, J, Hospital de Son Llatzer, Palma de Mallorca
- Conde, MLM, Hospital Arnau de Vilanova, Lleida
- Cruzado, JM, Hospital de Bellvitge, Barcelona
- Garcia N, Clinica Universitaria de Navarra, Navarra
- Goiciechea, M, Hospital Gregorio Marañon, Madrid
- Gonzales, F, Hospital Torrecardenas, Almeria
- I Porras, AB, Hospital del Mar, Barcelona
- Macia, M, Hospital de la Candelaria, Tenerife
- Marques M, Hospital Puerta de Hierro, Madrid
- Monzó, JJB, Hospital Clinic, Barcelona
- Munar, MA, Hospital Son Espaes, Palma de Mallorca
- Ortiz, A, Fundacion Jimenez Diaz, Madrid
- Saborido, MIS, Hospital Germans Trias I Pujoil, Barcelona
- Salgueira, M, Hospital Virgen Macarena, Sevilla
- Soler MJ, Hospital Vall d’Hebron, Barcelona
- Sancho, A, Hospital Universitario Dr. Peset, Valencia

For Singapore

- Liu YLA, Khoo Teck Puat Hospital, Singapore
- Teo BW, National University of Hospital System, Singapore
- Sreekanth K, Changi General Hospital, Singapore
- Lee WZI, SengKang General Hospital, Singapore
