# Supplementary material for: Rationale and design of the Renal Lifecycle trial assessing the effect of dapagliflozin on cardiorenal outcomes in severe chronic kidney disease
Source: Nephrol Dial Transplant. 2025 Mar 7;40(9):1746–55. doi: 10.1093/ndt/gfaf046 (PMC12394133; doi:10.1093/ndt/gfaf046)
Supplement: gfaf046_Supplemental_Files [file gfaf046_supplemental_files.zip › Supplementary 1- Informed consent form.docx]

**Appendix 1.**  Informed consent form in various languages. **A)** English version, **B)** German version) **C)** Dutch version.

**A) English version**

v. 5.0_27Feb2024

**A randomized controlled clinical trial to assess the effect of dapagliflozin on renal and cardiovascular outcomes in patients with severe chronic kidney disease
(The RENAL LIFECYCLE Trial)**

**Participant Information Sheet/Consent Form**

Interventional Study – Adult providing own consent

| **Australia Project Sponsor** | The George Institute for Global Health | **Global Project Sponsor** | University Medical Center Groningen |
| --- | --- | --- | --- |
| **Global Chief Investigator** | Professor Ronald Gansevoort | **Australian Chief Investigators** | Professor Sunil Badve and Associate Professor Clare Arnott |
| **Local Principal Investigator** | *[Insert PI Name]* | **Site/Hospital Name** | *[Insert Site name ]* |

You are invited to take part in a research study called The RENAL LIFECYCLE Trial. You are being asked to participate because you have been diagnosed with severe chronic kidney disease.

**Why are we doing this study?**

People with severe chronic kidney disease are at a risk of developing chronic kidney disease complications including kidney and heart failure. Research has shown that it may be possible to reduce the risk of these complications by using a medication called sodium glucose co-transporter 2 (SGLT2) inhibitor, which act in your kidneys to lower sugar levels in your blood. The aim of the RENAL LIFECYCLE Trial is to see whether the SGLT2 inhibitor, dapagliflozin, can slow the loss of kidney function and heart failure in patients with chronic kidney disease, including dialysis and kidney transplant patients.

**What does the study involve?**

- If you agree to take part in this study, the study team will invite you to attend a screening visit to check whether you are eligible to participate and if you are, you will be asked to sign the study Consent Form.
- You will then be randomised to the study. This means that you will be placed into one of two groups (dapagliflozin or placebo) using chance (like tossing a coin).


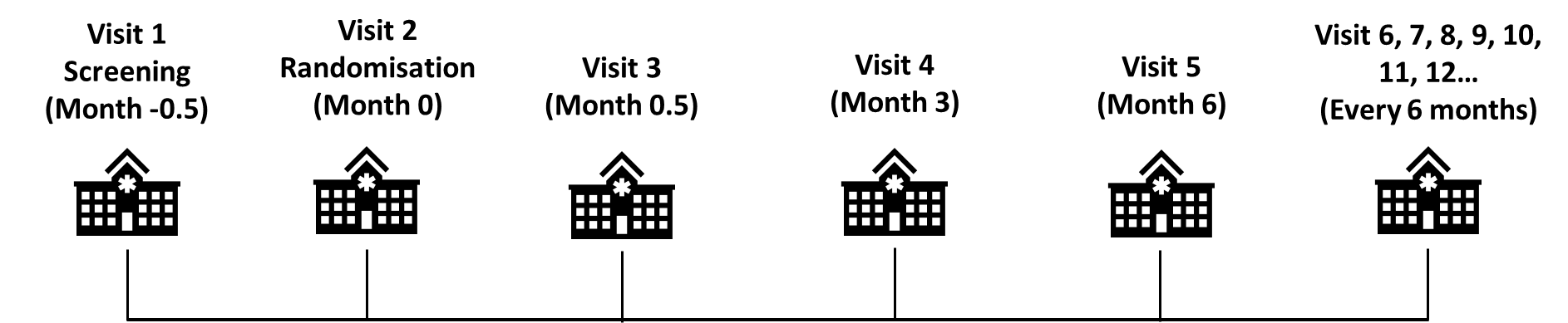
From your randomisation visit, you will be required to take dapagliflozin or placebo (depending on the group you are placed in) once a day, for the duration of the study (about 4 years). After the randomisation visit, you will be followed-up at two weeks, three months, six months and six monthly thereafter: all visits are face-to-face at your hospital and can often be combined with your normal hospital visits. Most visits will take a maximum of one hour. An overview of the study visits is outlined in the image below:

You are also invited to undergo an optional MRI (magnetic resonance imaging) and an echocardiogram of your heart, to see the effect of the medication on your heart. The MRI will be taken at a scanning centre located within an hour’s drive of your hospital. This scanning center has been selected and engaged by the Sponsor to perform these tests. The echocardiogram will be completed in your hospital during your study visit. You are also invited to complete an optional cognitive function test to see the effect of the medication on your thinking and movement speed. This test is completed at your hospital during your study visit.

Please note you can leave the study at any time if you or your doctor/medical specialist want you to. Your choice will not affect the care you get from your doctors and nurses.

**What are the risks?**

All medical treatments involve some risk of harm. The common known risks of dapagliflozin include genital yeast infection (thrush), passing more urine, headache and backpain. Uncommon, rare and very rare side effects include dehydration, diabetes ketoacidosis (the body produces excess blood acids, called ketones) and necrotising fasciitis (soft tissue infection of the genitals). If you get put in the placebo group, the chance of heart failure or kidney failure might be higher. You will be monitored closely by your study doctor to minimise this risk. There are more details about the risks in the following document.

**How will my confidentiality be protected?**

All information collected from you will be treated confidentially and stored securely. In any study reports or publications your identity will remain confidential.

# Table of Contents

[Table of Contents 2](#_Toc160008442)

[1 Introduction 3](#_Toc160008443)

[2 What is the purpose of this research? 3](#_Toc160008444)

[3 What does participation in this research involve? 4](#_Toc160008445)

[4 What do I have to do? 7](#_Toc160008446)

[5 Will I be paid? 8](#_Toc160008447)

[6 What are the possible benefits of taking part? 8](#_Toc160008448)

[7 What are the possible risks and disadvantages of taking part? 8](#_Toc160008449)

[8 What will happen to my test samples? 10](#_Toc160008450)

[9 What if new information arises during this research study? 10](#_Toc160008451)

[10 Can I have other treatments during this research study? 10](#_Toc160008452)

[11 Do I have to take part in this research study? 10](#_Toc160008453)

[12 What are the alternatives to participation? 11](#_Toc160008454)

[13 Can I withdraw from the study? 11](#_Toc160008455)

[14 Could the study be stopped unexpectedly? 11](#_Toc160008456)

[15 What happens when the research study ends? 11](#_Toc160008457)

[16 Confidentiality/Privacy 12](#_Toc160008458)

[17 What happens with the results? 12](#_Toc160008459)

[18 Compensation for injuries or complications 12](#_Toc160008460)

[19 Who is organising and funding the research? 13](#_Toc160008461)

[20 COVID-19 13](#_Toc160008462)

[21 Further information and who to contact 13](#_Toc160008463)

# Introduction

You are invited to take part in a research study called The RENAL LIFECYCLE Trial. You are being asked to participate because you have been diagnosed with severe chronic kidney disease. The research study is testing a new treatment for reducing the occurrence of kidney and heart failure in people with severe chronic kidney disease, including dialysis and kidney transplant patients. The treatment is called dapagliflozin (Forxiga®), a medication usually used in people with diabetes. This medication is known as a sodium glucose co-transporter 2 (SGLT2) inhibitor.

This Participant Information Sheet/Consent Form tells you about the research study. It explains the tests and treatments involved. Knowing what is involved will help you decide if you want the participant to take part in the research.

Please read this information carefully. Ask questions about anything that you don’t understand or want to know more about. Before deciding whether or not to take part, you might want to talk about it with a relative, friend or your usual doctor.

Participation in this research is voluntary. If you do not wish to take part, you don’t have to. You will receive the best possible care whether or not you take part.

If you decide you want to take part in the research study, you will be asked to sign the consent section. By signing it you are telling us that you:

Understand what you have read

Consent to taking part in the research study

Consent to having the tests and treatments that are described

Consent to the use of your personal and health information as described.

You will be given a copy of this Participant Information Sheet and Consent Form to keep.

# What is the purpose of this research?

Chronic kidney disease is a common illness, affecting 10% of the adult population worldwide. The most common causes of chronic kidney disease are diabetes, hypertension, and chronic glomerulonephritis (the long-term inflammation and scarring of the glomeruli, which are tiny blood vessels in the kidney that filter blood and produce urine). People living with chronic kidney disease are at high risk of serious complications such as kidney failure and heart failure.

Current treatment of chronic kidney disease includes blood pressure control as well as blood sugar control in people who also have type 2 diabetes. This helps to reduce the risk of developing chronic kidney disease complications. These treatments have been successful, but there remains a high number of people with chronic kidney disease who develop kidney failure and heart failure. Research has shown that it may be possible to reduce the risk of these complications by using an additional medication for people who develop severe chronic kidney disease, called a sodium glucose co-transporter 2 (SGLT2) inhibitor.

SGLT2 inhibitors act in your kidneys, causing you to lose more sugar into your urine, which lowers the sugar level in your blood. They may also reduce your chance of developing a serious kidney or heart problem.

Research conducted in people with chronic kidney disease often excluded people with poor renal function (i.e., estimated glomerular filtration rate [eGFR] less than 25ml/min/1.73m^2^, dialysis patients and renal transplant recipients). People with poor renal function are at higher risk of kidney failure, heart failure and death. New research has shown that SGLT2 inhibitors may be effective in preventing kidney failure, heart failure and death in people with severe chronic kidney disease, including dialysis and renal transplant patients.

The RENAL LIFECYCLE trial is testing whether the SGLT2 inhibitor, dapagliflozin, can slow the loss of kidney function and heart failure in patients with severe chronic kidney disease, including dialysis and kidney transplant patients. We are also investigating the safety and how well you tolerate this drug.

Dapagliflozin is approved in Australia to treat type 2 diabetes, symptomatic heart failure with reduced ejection fraction (when your heart pump function is impaired) and in patients with chronic kidney disease (stage 2, 3, or 4) who are at risk of disease progression. However, it is not currently approved to treat people with severe chronic kidney disease who have an eGFR less than 25mL/min/1.73m^2^. Therefore, this study is an experimental treatment for dapagliflozin. This means that it must be tested to see if it is an effective treatment for people with severe chronic kidney disease, including dialysis and kidney transplant patients. This study will be conducted under the Therapeutic Goods Administration (TGA) Clinical Trials Notification (CTN) Scheme. This allows the investigators to use this product for medical research purposes once the research has been assessed and approved by an authorised Human Research Ethics Committee (HREC).

This study is being conducted at 10-15 hospitals across Australia and in multiple sites across Europe. Approximately 1500 participants will be recruited for this study from Australia, The Netherlands, Germany, and Belgium. In Australia, we expect 250 participants will join the study.

This research has been initiated by Professor Ronald Gansevoort at the University Medical Center, Groningen (UMCG) in the Netherlands in collaboration with a team of doctors and researchers based at national and international healthcare facilities and universities. In Australia, this research is being conducted by Professor Sunil Badve and Associate Professor Clare Arnott at the George Institute for Global Health, an affiliate of the University of New South Wales, Australia.

This research has been funded in Australia by a National Health and Medical Research Council (NHMRC) 2021 Medical Research Future Fund (MRFF) International Clinical Trial Collaborations (ICTC 21-1 Application 2015414).

# What does participation in this research involve?

If you decide to participate in this study, you will be participating in a randomised controlled trial. Sometimes we do not know which treatment is best for treating a condition. To find out we need to compare different treatments. We put people into groups and give each group a different treatment. The results are compared to see if one is better. To try to make sure the groups are the same, each participant is put into a group by chance (i.e., random).

This is a double-blind study. This means that you and the study doctor will not know which treatment you are receiving. However, in certain circumstances the study doctor can find out which treatment you are receiving. You will have an equal chance of receiving either the study medication (i.e., dapagliflozin) or a placebo. A placebo is a medication with no active ingredients. It looks like the real thing but is not. Each group will continue to receive standard care regardless of their treatment arm.

The study medication will be provided to you free of charge.

After the randomisation visit, you will be followed-up at two weeks, three months, six months and six monthly thereafter: all visits are face-to-face at your hospital and can often be combined with your normal hospital visits. Your participation in the study will last about 48 months (i.e., 4 years). However, the exact duration of the study may be shorter or longer than the intended 48 months, depending on, among other things, how quickly the total number of required participants can be included. Most visits will take a maximum of one hour.

The consent form (attached to this information sheet) must be signed before any study procedures or visits take place.

All study visits will take place at your hospital, with a member of the study team.

## Visit 1 – Screening Visit (-2 weeks)

If a study hospital staff team member thinks that this study may be suitable for you to take part in, you will be invited to attend a screening visit.

After you have signed the informed consent form the following screening tests will then be performed to check your suitability for the trial:

- You will be asked questions about your lifestyle, medical history, any medications you are taking.
- Measurement of blood pressure, heart rate, height, and weight.
- You will be asked to complete blood and urine tests.
- Blood or urine pregnancy test for people of child-bearing potential.

We may need more information from your general practitioner/medical specialist. We will only do this if it is in connection with your own safety. Examples of when we ask for more information are when your medication use is not completely clear or when we need more information about surgeries and/or treatment methods you have had in the past. We will inform you before we contact your general practitioner and/or treating medical specialist.

If the results of the screening tests indicate that the study is not suitable for you, you may be eligible to repeat the screening tests after one month at the study doctor’s judgment. If you are eligible to rescreen you will need to sign a new consent form and you will receive a new participant ID. If you meet all entry criteria, you can proceed to the randomisation visit.

If the results of the screening tests indicate that the study is not suitable for you, you will not be able to take part. If this is the case, the study coordinator will discuss this with you and arrange for your regular health care to continue.

## Assessments completed at each study visit

- Review of your health since the last visit and if there have been any changes to your medications.
- Measurement of blood pressure, heart rate, and weight.
- You will be asked to complete a blood test.

## In addition, the following assessments will be completed at:

## Visit 2 – Randomisation Visit – Day 0

- You will be asked to complete a urine test.
- You will be asked to complete two questionnaires to assess your quality of life and your general health.
- You will have the option to complete two scans – a cardiac MRI and/or echocardiogram.
- You will have the option to complete a cognitive function test.

## Visit 3 – Week 2

- How many pills of the study medication you have taken since your last visit and if you have experienced any side effects. You will be asked to return any unused study medication including empty containers.

## Visit 4 – Month 3

- How many pills of the study medication you have taken since your last visit and if you have experienced any side effects. You will be asked to return any unused study medication including empty containers.

You will be asked to complete a urine test.

## Visit 5 – Month 6

- How many pills of the study medication you have taken since your last visit and if you have experienced any side effects. You will be asked to return any unused study medication including empty containers.

You will be asked to complete a urine test.

- You will be asked to complete two questionnaires to assess your quality of life and your general health.
- You will have the option to complete one scan – an echocardiogram.
- You will have the option to complete a cognitive function test.

## Visit 6 Onwards – 6-Monthly Visits

- How many pills of the study medication you have taken since your last visit and if you have experienced any side effects. You will be asked to return any unused study medication including empty containers.

You will be asked to complete a urine test.

- You will be asked to complete two questionnaires to assess your quality of life and your general health every 12 months.
- You will have the option to complete two scans – a cardiac MRI and/or echocardiogram at your 12-month visit.
- You will have the option to complete a cognitive function test every 12 months.

## End of Study or Early End of Treatment Visit

- You will be asked to complete a urine test.
- You will be asked to complete two questionnaires to assess your quality of life and your general health.
- You will have the option to complete a cognitive function test.

## Information About Your Blood Tests and Urine Tests Throughout the Study

- Blood tests:
  - You will be asked to fast (to not eat or drink anything apart from water for 8 hours) before the blood tests.
  - At your screening, 2 weeks, 3 months, 6 months and 6 monthly thereafter visits - The blood tests will involve collection of 20 mL (or 4 teaspoons) of blood to measure your kidney function.
  - At your randomisation and end of study/early end of treatment visits - The blood tests will involve collection of 20 mL (or 4 teaspoons) of blood and will measure your kidney function, blood sugar, cholesterol, calcium and phosphorus.
  - At your randomisation, 3 month and end of study/early end of treatment visits – you will have the option to provide an additional 20mL (or 4 teaspoons) of blood for storage, this is so additional tests can be done after the study.
- Urine tests:
  - The urine test will involve collection of 40-50 mL of urine and will measure your kidney health.
  - Urine collection takes place in the morning of the study visit.
  - At the randomisation, 3 months, 6 months and 6 monthly thereafter visits - Participants on dialysis will need to collect urine for 24 hours.
  - At your randomisation, 3 month and end of study/early end of treatment visits – you will have the option to provide an additional 40-50mL of urine for storage, this is so additional tests can be done after the study.

## Unscheduled Visit

If you decide to stop participating at any stage throughout the study before the final study visit, or need to come in for any reason, you will be asked to return to your hospital for a visit. The Early End of Treatment Visit procedures described above will be completed during this visit.

## Important Information About Your Study Medication

The dose of study medication you receive at this visit and throughout the study will be the standard dose that is approved for the treatment of chronic kidney disease in Australia, but you will not know if you are taking the study medication or a placebo. You will take one pill of study medication per day. The study medication should be stored at room temperature and away from light, excess heat, and moisture (not in the bathroom). The study medication should be taken once a day in the morning with a little water (around the same time every day between 7 and 9 o’clock in the morning). It doesn’t matter if you take the drug before or after breakfast.

**If you forget to take your study medication** and it has been less than 6 hours since you usually take it, take your study medication immediately. If it has been more than 6 hours, take your next dose of study medication at the usual time the following day. Do not take a double dose to make up for the dose that you missed.

**If you require a planned surgery,** it is necessary that you temporarily stop taking your study medication at least three days before your surgery. If you require an emergency surgery, the surgery can proceed and your doctors will monitor you closely after the surgery for any side effects. Your study medication can be re-started once you are eating and drinking as usual or as instructed by your doctors.

If you are unwell and attend a healthcare facility e.g., GP Practice or Hospital Emergency Department, please show them your RENAL LIFECYCLE Wallet Card, as this will explain that you are taking part in a clinical trial and may be taking Dapagliflozin or placebo.

## Optional Assessments

### Cardiac MRI Scan

You are also invited to undergo a MRI (magnetic resonance imaging) scan of your heart. The study researchers are looking at the effect of the study medication on your heart. These scans will be taken at a scanning centre located within an hour’s drive of your hospital. This scanning center has been selected and engaged by the Sponsor to administer these tests. If you agree to participate, your identifying information (e.g. name and contact details) will be forwarded to the MRI provider for the purpose of booking your appointment.

An MRI scanner is a machine that uses electromagnetic radiation (radio waves) in a strong magnetic field to take clear pictures of the inside of the body. Electromagnetic radiation is not the same as ionising radiation used, for example, in X-rays. The pictures taken by the machine are called MRI scans.

You will be weighed before being asked to lie on a table inside the MRI scanner. You’ll be asked to lie in a comfortable position and stay still for around 15 minutes. The scanner will create images of your heart. The scanner is very noisy, you may be given some earphones to reduce the noise. Some people may experience symptoms of claustrophobia from lying in a confined space. If you do experience discomfort at any time during the scan, you will be able to alert staff by pressing on a call button provided to them.

There are no proven long-term risks related to MRI scans as used in this research study. MRI is considered to be safe when performed at a centre with appropriate procedures. However, the magnetic attraction for some metal objects can pose a safety risk, so it is important that metal objects are not taken into the scanner room.

You will be thoroughly examined to make sure there is no reason to not have the scan. You must tell study staff if you have metal implanted in your body, such as a pacemaker or metal pins.

The scans we are taking are for research purposes. They are not intended to be used like scans taken for a full clinical examination. The scans will not be used to help diagnose, treat or manage a particular condition. A specialist will look at your MRI scans for features relevant to the research study. On rare occasions, the specialist may find an unusual feature that could have a significant risk to your health. If this happens, we will contact you to talk about the findings. We cannot guarantee that we will find any/all unusual features.

If required, you may be reimbursed for any additional travel or parking expenses associated with the cardiac MRI visits .

### Echocardiogram

You are also invited to undergo an echocardiogram of your heart. The study researchers are looking at the effect of the study medication on your heart. This test will be completed in your hospital during your study visit. An echocardiogram is a painless test looking at the electrical signals of your heart. Electrodes will be stuck to various parts of your chest to measure your heart rate and normality of your heartbeat. This assessment will take around 15 mins.

If you opt into doing the echocardiogram, you will also be asked to complete a questionnaire on your heart health, this should take around 5-10 minutes.

### Cognitive function

You are also invited to complete a cognitive function test. The study researchers are looking at the effect of the study medication on your thinking and movement speed. This test will be completed in your hospital during your study visit. The test is completed on a mobile phone or tablet and takes 90 seconds.

# What do I have to do?

If you decide to participate:

- You need to attend the scheduled visits and cooperate with the study procedures as described in Section 3 above.
- You must tell your study coordinator if you have participated in another research study in the past year or are currently in another research study. While participating in this study, you should not take part in another research study without approval from your doctor.
- You must carry the participant card of the study with you, for example in your wallet. The card states that you are participating in this study and whom to warn in the event of an emergency. Show this card when you visit a (other) physician.
- You need to inform the study coordinator about any health problems, accidents or medical interventions that happen while you are in the study, even if you think it is not important.
- You need to inform the study coordinator if you have been taking or not taking your study medication.
- You need to inform the study coordinator if you start any new medication or stop any medication that you are already taking. This includes prescribed or over the counter products.
- You must remember to bring your unused study medication and all empty containers to each of your study visits and explain if there is any lost or missing study medication.
- You need to inform the study coordinator if you decide not to continue in the study. You don’t have to give a reason for your decision.
- There are no lifestyle restrictions (e.g., physical restrictions, participation in sport) or dietary restrictions in this study.

# Will I be paid?

All medication, tests and medical care required as part of the research study will be provided to you free of charge. There are no additional costs associated with participating in this research study, nor will you be paid.

# What are the possible benefits of taking part?

We cannot guarantee or promise that you will receive any benefits from taking part in this study; however, possible benefits may include a reduction in the risk of heart complications and slowing the progression of kidney disease. Some people may also benefit from the information learned in this study. This research may help to develop a new therapy for others with similar conditions.

# What are the possible risks and disadvantages of taking part?

Medical treatments often cause side effects. You may have none, some, or all the effects listed below, and they may be mild, moderate, or severe. If you have any of these side effects, or are worried about them, talk with your GP/ treating medical specialist or the study coordinator. Your GP/treating medical specialist and the study coordinator will also be looking out for side effects. It is very important that you tell your GP/treating medical specialist and the study coordinator about your full medical history and about all the medications you are taking as some medical conditions and medications may mean it is not suitable for you to take the study medication.

There may be side effects that the researchers do not expect or do not know about and that may be serious. Tell your GP/treating medical specialist and the study coordinator immediately about any new or unusual symptoms that you get.

If you experience stomach upset, including vomiting or diarrhoea or an infection, and/or you are unable to eat, let your study coordinator know as soon as possible as it may be necessary to temporarily stop taking your study medication until you are feeling better.

If you require an admission to hospital or surgical procedure/operation, or a scan with intravenous contrast (e.g., CT scan), let your and study coordinator know as soon as possible as it may be necessary to temporarily stop taking your study medication (please see section ‘Important Information About Your Study Medication’ in this Participant Information Sheet and Consent Form for further information).

Many side effects go away shortly after treatment ends. However, sometimes side effects can be serious, long lasting, or permanent. If a severe side effect or reaction occurs, you may need to stop your treatment. Your GP/treating medical specialist will discuss the best way of managing any side effects with you.

The risks associated with dapagliflozin are well known.

*For Diabetic Participants –* Dapagliflozin does not normally cause hypoglycaemia (low blood sugar) although hypoglycaemia may occur when dapagliflozin is taken with other medications to lower blood sugar levels e.g., sulfonylurea or insulin. Symptoms of hypoglycaemia may appear suddenly and include feeling weak and/or shaky, light-headedness, dizziness, inability to concentrate, a fast or pounding heartbeat, sweating and hunger. If you experience these symptoms, it is important to raise your blood sugar levels by eating 5 to 7 jellybeans, 3 teaspoons of sugar or honey, or drinking half a can of full sugar soft drink. You should also inform your GP/medical specialist and study coordinator as soon as possible.

**Dapagliflozin**

*Common side effects (occur in between 1 in 10 and 1 in 100 people)*

- 1. Genital yeast infection (thrush): symptoms include genital burning, redness, pain, and discharge.
  2. Passing more urine than usual or need to urinate more often
  3. Headache
  4. Back pain

The side effects listed above are usually mild and short-lived.

*Uncommon side effects (occur in between 1 in 100 and 1 in 1,000 people)*

- 1. Dehydration: symptoms include unusual thirst, light-headedness, or dizziness upon standing, and fainting or loss of consciousness. If you experience these symptoms, contact your GP and the study coordinator as soon as possible.

*Rare side effects (occur in between 1 in 1,000 and 1 in 10,000 people)*

- 1. Diabetes ketoacidosis (also known as DKA) is a rare but very serious possible side effect of dapagliflozin. Symptoms include vomiting, abdominal/stomach pain, excessive thirst, deep and fast breathing, confusion, unusual sleepiness or tiredness, sweet smelling breath, rapid weight loss. If you experience these symptoms, stop taking your study medication immediately and attend your nearest emergency department. You should also inform your GP and the study coordinator as soon as possible.

*Very rare side effects (occurs in less than 1 in 10,000 people)*

- 1. Necrotising fasciitis of the perineum or Fournier’s gangrene is a very rare, serious soft tissue infection of the genitals or the area between the genitals and the anus. Symptoms include pain or tenderness, itching, swelling in the genital or back passage area, fever or generally feeling unwell. If you experience these symptoms, stop taking your study medication immediately and attend your nearest emergency department. You should also inform your GP and the study coordinator as soon as possible.

Some side effects may occur that usually do not need medical attention. These side effects may go away during treatment as your body adjusts to the medicine. Also, your GP may be able to tell you about ways to prevent or reduce some of these side effects.

**Pregnancy Risk**

The effects of dapagliflozin could harm an unborn child. It is not safe to take this medicine during the second and third trimester of pregnancy. Because of this, it is important that research participants are not pregnant or breast-feeding and do not become pregnant during the research study. You must not participate in the research if you are pregnant or trying to become pregnant, or breast-feeding. If you are person of childbearing potential there is a possibility, you will be required to undergo a pregnancy test prior to commencing the research study.

Participants of childbearing potential are strongly advised to use effective contraception during the research and for a period of three months after completion of the research study. You should discuss methods of effective contraception with your study doctor.

If you do become pregnant whilst participating in the research study, you should advise the study coordinator and your GP immediately. You will be withdrawn from the research study and advise on further medical attention should this be necessary. You must not continue in the research if you become pregnant.

**Blood Tests**

Having a blood sample taken may cause some discomfort, bruising, minor infection, or bleeding. If this happens, it can be easily treated.

# What will happen to my test samples?

Blood and urine samples will be collected at your hospital by the study coordinator for standard safety evaluations and will be tested at your hospital laboratory. These samples will be labelled with your name and date of birth, as they would be if you were not in a research study and linked to your medical record as per any standard blood and urine test. Your test results will be entered into the study database by the study coordinator in a de-identified form meaning they will not contain your name, only your study participant number. Access to your information will be strictly controlled and only authorised research staff members will have access. Following this, these samples will be securely destroyed after testing, according to the hospital laboratory’s standard procedures. All hospital laboratories will be accredited organisations to perform human blood and urine testing.

Some Australian hospitals are participating in the donation of additional blood and urine samples for future analysis. If your hospital is participating, your study coordinator will discuss this with you. Your donation of these additional samples is optional. You can still participate in the study and not provide additional samples.

If you choose to participate you will be asked to provide additional consent, to indicate whether you agree to the collection and storage of your blood and urine samples. The purpose of collecting these samples is to explore if there are associations with severe chronic kidney disease, effects of the study medication, clinical outcomes, and toxicity. These samples will be collected by your hospital pathology services, spun/prepared using standard operating procedures and stored in the freezer facility of the local pathology department at your hospital in Australia. Samples will then be transferred to the University Medical Center Groningen, Netherlands and stored for a maximum of 25 years, after which they will be destroyed. These additional samples will be labelled with your study participant number, visit number and date. The list linking your study participant number to your name will be stored at a secure place in the hospital. Only participant numbers will be used, to process your additional blood and urine samples. Access to your information will be strictly controlled and only authorised research staff members will have access.

# What if new information arises during this research study?

Sometimes during a research study, new information becomes available about the treatment that is being studied that may affect your willingness to continue in the study. If this happens, your study team will tell you about it in a timely manner and discuss with you whether you want to continue in the research study. If you decide to withdraw, your GP/treating medical specialist will arrange for your regular health care to continue. If you decide to continue in the research study, you will be asked to sign an updated consent form.

On receiving new information, your GP/treating medical specialist might consider it to be in your best interests to withdraw you from the research study. If this happens, they will explain the reasons and arrange for your regular health care to continue.

# Can I have other treatments during this research study?

Whilst you are participating in this research study, your GP/treating medical specialist will continue to manage your clinical care and medications as per usual practice.

It is important to tell your GP/treating medical specialist and the study coordinator about any treatments or medications you may be taking, including over-the-counter medications, vitamins or herbal remedies, acupuncture, or other alternative treatments. You should also tell your GP/treating medical specialist and the study coordinator about any changes to these during your participation in the study. Your GP/treating medical specialist should also explain which of your treatments or medications need to be stopped for the time you are involved in the study.

It may also be necessary for you to take medication during or after the research study to address side effects or symptoms that you may have. You may need to pay for these medications and so it is important that you ask your doctor about this possibility.

# Do I have to take part in this research study?

Participation in any research study is voluntary. If you do not wish to take part, you do not have to. If you decide to take part and later change your mind, you are free to withdraw from the study at any stage.

If you do decide to take part, you will be given this Participant Information Sheet and Consent Form to sign and you will be given a copy to keep.

Your decision whether to take part or to take part and then withdraw, will not affect your routine treatment, your relationship with those treating you or your relationship with your hospital.

# What are the alternatives to participation?

You do not have to take part in this research study to receive treatment at your hospital. You may continue your usual care for your severe chronic kidney disease without being in this research study or you may be able to take part in another research study. Your study hospital staff team member will discuss these options, including their possible benefits and risks, with you before you decide whether to take part in this research study. You can also discuss the options with your general practitioner (GP), treating medical specialist and other treating healthcare professionals.

# Can I withdraw from the study?

If you decide to withdraw from the study, please notify a member of the study team beforehand. This notice will allow the study coordinator or your GP/treating medical specialist to ask you your reason for withdrawing and discuss any health risks or special requirements linked to withdrawing.

You may withdraw from taking the study medication or withdraw your consent to participate in the study. If you do withdraw your consent during the study, you will be asked to complete one final visit. If you decide to withdraw, you will have the option to allow study staff at the end of the study to contact you, your GP/treating medical specialist, or another party (this includes publicly available sources) to obtain information about your health status.

If you do decide to withdraw, study staff will not collect additional personal information from you, although personal information already collected will be retained to ensure that the results of the research study can be measured properly and to comply with law. You should be aware that data collected by the sponsor up to the time you withdraw will form part of the study results. Please let us know at the time when you withdraw what you would like us to do with the information we have collected up to that point.

# Could the study be stopped unexpectedly?

Your participation in the study may be stopped without your consent at any time. The reasons may include:

- You have side effects from study treatment and further study treatment is not in your best interest;
- Your medical condition changes and further study treatment is not in your best interest;
- You do not attend clinic visits and cooperate in the study procedures as described in Section 3 of this Participant Information Sheet;
- Study procedures are not followed, and continued study participation is not in your best interest;
- A decision by health authorities in Australia or overseas; or
- The study is cancelled by the trial global sponsor, UMCG or the local sponsor, The George Institute for Global Health.

This research study may be stopped unexpectedly for a variety of reasons. These may include reasons such as:

- Unacceptable side effects
- The drug/treatment being shown not to be effective
- The drug/treatment being shown to work and not need further testing

Decisions made in the commercial interests of the sponsor or by regulatory/health authorities.

# What happens when the research study ends?

The study medication, dapagliflozin, is not yet proven for use in people with poor renal function (eGFR less than 25ml/min/1.73m^2^). Therefore, you will not be able to continue to receive dapagliflozin indefinitely. When the study ends, your GP/treating medical specialist will discuss treatment choices with you.

You have a right to receive feedback about the overall results of this study. You can tell us that you wish to receive feedback by ticking the relevant box on the Participant Consent Form. A study team member will provide you with a summary of the results of the study. The study team member can also tell you which treatment you received.

# Confidentiality/Privacy

Any identifiable information that is collected about you in connection with this study will remain confidential and will be disclosed only with your permission, or except as required by law. Only the study researchers, monitors, representatives of regulatory authorities and ethics committee may have direct access to it. Access is required to check the accuracy of the information collected and to ensure that this trial is being carried out according to local requirements and/or regulatory guidelines.

Study monitors, auditors, representatives of regulatory authorities and ethics committee may also be granted direct access to your original medical records for verification of trial procedures and/or data.

All electronic information collected will be entered directly into a secure web-based database hosted by the global sponsor in The Netherlands - University Medical Center, Groningen. All electronic information will be deidentified to protect your confidentiality and computer records will be password-protected. Information collected from you using paper-based measures will be deidentified and stored securely at your hospital (with later transfer at the end of the study to The George Institute for Global Health for secure storage), and only approved research personnel will have access to this information. For those who complete the cardiac MRI sub-study, electronic images will be transferred directly to The George Institute for Global Health for storage on our password and firewall protected server in a deidentified form. All deidentified electronic images will then be securely transferred to Mycardium AI in the United Kingdom for data analysis. All electronic and paper-based trial documentation will be kept and securely archived for 25 years. After this time, it will be securely destroyed.

You will be asked to provide your consent for the research team to share or use the information collected from you in future, ethics-approved research that:

- Will be specific to the aims of this research; and/or
- Will be used in any future research.

Your information will only be shared in a format that will not identify you.

# What happens with the results?

All information collected from you for this study will be stored electronically in a database maintained by UMCG. It is intended for the results of this study to be presented or published at medical conferences and in scientific journals.

In any publication, information will be provided in such a way that you cannot be identified. Results of the study will be provided to you, if you wish. By signing the consent form, you agree to your data being included in the results published for this study.

A description of this clinical trial will be available on [www.clinicaltrials.gov](http://www.clinicaltrials.gov). This website will not include information that can identify you. At most, the website will include a summary of the results. You can search this website anytime.

# Compensation for injuries or complications

If you suffer any injuries or complications as a result of this study, you should contact your GP as soon as possible, who will assist you in arranging appropriate medical treatment. If you are eligible for public health care or medical insurance, you can receive any medical treatment required to treat the injury or complication, free of charge, as a public patient in any public hospital.

In addition, you may have the right to take legal action to obtain compensation for any injuries or complications resulting from the study. Compensation may be available if your injury or complication is sufficiently serious and is caused by unsafe drugs or equipment, or by negligence of one of the parties involved in the study (for example, the researcher, or the treating doctor). You do not give up any legal rights to compensation by participating in this study.

# Who is organising and funding the research?

The study is being funded in Australia by a NHMRC 2021 MRFF International Clinical Trial Collaboration Grant. The study medication is being provided in-kind by the pharmaceutical company AstraZeneca who make dapagliflozin. AstraZeneca has had no role in the design and conduct of the study, and will not be involved in interpretation of results and publication of data.

You will not benefit financially from your involvement in this research study even if, for example, knowledge acquired from analysis of your samples prove to be of commercial value to the UMCG and/or The George Institute for Global Health.

In addition, if knowledge acquired through this research leads to discoveries that are of commercial value to the UMCG, The George Institute for Global Health or the study hospitals there will be no financial benefit to you or your family from these discoveries.

No member of the research team will receive a personal financial benefit from your involvement in this research study (other than their ordinary wages).

# COVID-19

Considering the COVID-19 pandemic, The George Institute for Global Health would like to alert participants that all sites will be working to the national guidelines which may involve confirming participants’ health status prior to their scheduled visit and rescheduling visits if you are sick with the flu or COVID-19. At your visit, the site will request your COVID-19 status.

# Further information and who to contact

When you have read this information, the study doctor and/or study coordinator will discuss it with you further and answer any questions you may have. If you would like to know more at any stage, please feel free to contact:

| **Site Principal Investigator:** |  |
| --- | --- |
|  | *[Insert site-specific details]* |
| **Complaints:** |  |
|  | *[Insert site-specific procedures]* |

## Ethics Approval

All research involving humans is reviewed by an independent group of people called a Human Research Ethics Committee (HREC) or Institutional Review Board (IRB). This study has been approved by the Ethics Review Committee (RPAH Zone) of the Sydney Local Health District. Any person with concerns or complaints about the conduct of this study should contact the Executive Officer on 02 9515 6766 and quote protocol number X22-0201.

The conduct of this study at the [*name of hospital*] has been authorised by the [*name of Local Health District*]. Any person with concerns or complaints about the conduct of this study may also contact the Research Governance Officer [*or other officer*] on [*telephone number*] and quote protocol number [*insert local protocol number*].

This study will be carried out in accordance with the *National Statement on Ethical Conduct in Human Research (2007, updated May 2018)*. This statement has been developed to protect the interests of people who agree to participate in human research studies.

**Thank you for taking the time to consider this study. If you wish to take part in it, please sign the attached consent form. This information sheet is for you to keep.**

**Consent Form**

Interventional Study – Adult providing own consent

| **Title** | A randomized controlled clinical trial to assess the effect of dapagliflozin  on renal and cardiovascular outcomes in patients with severe chronic kidney disease |
| --- | --- |
| **Short Title** | The RENAL LIFECYCLE trial |
| **Global Study Sponsor** | University Medical Center Groningen |
| **Australia Study Sponsor** | The George Institute for Global Health |
| **Global Chief Investigator** | Professor Ronald Gansevoort |
| **Australian Chief Investigators** | Professor Sunil Badve and Associate Professor Clare Arnott |
| **Local Principal Investigator** | *[Insert PI Name]* |
| **Location** | *[Location]* |

**Declaration by Participant**

I, __________________________________________________________________________

*[Print full name]*

have read and understood the Information for Participants on the above-named research study.

1. I have been made aware by __________________________________ (“the researcher”) of the procedures involved in the study, time involved, including any known or expected inconvenience, risks, discomfort or potential side-effects and of their implications as far as they are currently known.
2. I understand that the researcher will conduct this study in a manner conforming to ethical and scientific principles set out by the National Health and Medical Research Council (NHMRC) of Australia and the Good Clinical Research Practice Guidelines of the Therapeutic Goods Administration.

1. I acknowledge that I have been given time to consider the information and to seek other advice.
2. I acknowledge that refusal to take part in this study will not affect the usual treatment of my condition.
3. I acknowledge that I am volunteering to take part in this study and I may withdraw at any time.
4. I understand that any blood and urine samples collected will only be used for this research study, as described in the relevant section of the Participant Information Sheet.
5. I acknowledge that this research has been approved by: the Sydney Local Health District Human Research Ethics Committee.
6. I acknowledge that any regulatory authorities may have access to my medical records concerning my disease and treatment for the purposes of this study. However, I understand my identity will not be disclosed to anyone else or in publications or presentations.
7. I give permission for my doctors, other health professionals, hospitals, or laboratories outside this hospital site to release information to my GP, hospital and the study coordinator concerning my disease and treatment for the purposes of this study. I understand that such information will remain confidential.
8. I understand that I may be contacted after the end of this study to be invited to participate further for assessment of my health and wellbeing in the longer term.
9. I understand that I will be given a signed copy of this document and the Participant Information sheet to keep.
10. I would like to receive a copy of the study results when they become available.
    My email address is:________________________________________________

| **Additional Consent** | **Yes or No** |
| --- | --- |
|  |  |
| 1. I consent to my coded data being used for future research. |  |
|  |  |
| 1. I consent to the collection and storage of additional blood and urine samples to use it for future research |  |
|  |  |
| 1. I consent to being approached after this study is finished to ask whether I want to participate in a follow-up study |  |
|  |  |
| 1. I consent to the researcher’s notifying me which treatment I have had after all participants have completed the study and this information becomes available   My email address is: ___________________________________________________ |  |
|  |  |
| 1. I consent to the use of my email address for digitally completing the questionnaires   My email address is: ___________________________________________________ |  |
|  |  |
| 1. I consent to participate in the **Cardiac MRI sub-study**. I understand that I may withdraw participation from the sub-study at any time without any affect on my participation in the main study.   *An MRI scan at baseline and 12 months.* |  |
|  |  |
| **For participants on peritoneal dialysis only**   1. I consent to participate in the **Echocardiogram sub-study**. I understand that I may withdraw participation from the sub-study at any time without any affect on my participation in the main study.   *An echocardiogram and questionnaire at baseline, 6 months and 12 months.* |  |
|  |  |
| 1. I consent to participate in the **Cognitive sub-study**. I understand that I may withdraw participation from the sub-study at any time without any affect on my participation in the main study.   *A cognitive function test at baseline, 6 months, 12 months and once every 12 months thereafter until end of study or early end of treatment.* |  |

| **Name of Participant** |  | | |  |
| --- | --- | --- | --- | --- |
|  | *(please print – First name / Family name)* | | |  |
| **Signature** |  | **Date** |  |  |
|  |  |  |  |  |

**Declaration by Witness**

I have witnessed and certify the Participant’s verbal consent to voluntarily agree to participate in this research study.

| **Signature of Impartial Witness** |  | | **Date** |  |  |
| --- | --- | --- | --- | --- | --- |
| *(to be completed only if the participant cannot read the participant information sheet)* | | | | |  |
| **Printed name of Impartial Witness** |  | **Relationship to the Participant** | |  |  |
| The Participant’s confirmation is attested by the above signature of an Impartial Witness | | | | |  |

**Declaration by Study Staff Member^†^**

I have given a verbal explanation of the research study; its procedures and risks and I believe that the participant has understood that explanation.

| **Name of Study Staff Member Delegated to Conduct Consent** |  | | |  |
| --- | --- | --- | --- | --- |
|  | *(please print – First name / Family name)* | | |  |
| **Signature** |  | **Date** |  |  |
|  |  |  |  |  |

^†^A senior member of research team must provide the explanation of, and information concerning, the research study. The staff member conducting consent must be delegated to do so on the study delegation log.

**Note: All parties signing the consent section must date their own signature.**

**B) German version**

V1.1_20Dec2022

**Randomisierte, kontrollierte klinische Studie zur Untersuchung der**

**Wirkung von Dapagliflozin auf die Nieren und das kardiovaskuläre**

**System bei Patienten mit schwerer chronischer Nierenerkrankung (RENAL LIFECYCLE Trial)**

EUDRACT-Nr. 2021-005446-15

Prüfstelle: <Adresse>

<PLZ, Stadt>

Prüfarzt: <Name>

<Telefon>

Name und Anschrift des Sponsors: University Medical Center Groningen

Hanzeplein 1, 9700 RB Groningen, Niederlande vertreten durch Prof. Dr. Ron T. Gansevoort

Leiter der klinischen Prüfung in Deutschland: Prof. Dr. med. Christoph Wanner

Universitätsklinikum Würzburg (DZHI)

Am Schwarzenberg 15, Haus A15

97080 Würzburg

# Kurzzusammenfassung

Sehr geehrte Patientin, sehr geehrter Patient,

- wir möchten Sie einladen, an einer klinischen Studie namens RENAL LIFECYCLE Trial teilzunehmen. Der Kurztitel der Studie lautet: Studie zum Herz- und Nierenschutz mit Dapagliflozin bei Patienten mit schwerer Nierenerkrankung.
- Die Entscheidung, an der Studie teilzunehmen, ist freiwillig.
- Im Rahmen dieser Studie wird untersucht, ob sich durch die Einnahme des Medikaments Dapagliflozin das Risiko einer Verschlechterung der Nierenerkrankung bzw. der Herzerkrankung bei Patienten mit einer bestehenden schweren Nierenerkrankung verringert.
- Dapagliflozin, ein sogenannter SGLT2-Hemmer (Sodium dependent glucose co-transporter 2), wird bereits zur Behandlung von Diabetes, Herz-Kreislauf-Erkrankungen und Erkrankungen der Nieren eingesetzt.
- In den bisherigen Studien wurden jedoch keine Patienten mit schlechter Nierenfunktion (eGFR < 25 ml/min/1,73 m2, Dialyse-, und transplantierte Patienten) untersucht. Daher wollen die Wissenschaftler der Universität Groningen, Niederlande, in dieser Studie untersuchen ob Dapagliflozin eine Verschlechterung der Nierenerkrankung oder den Tod infolge einer Herzerkrankung bei Patienten mit einer schweren Nierenerkrankung verhindern kann.
- Wenn Sie sich für die Teilnahme an der Studie entscheiden, werden Sie gebeten, in den ersten 6 Monaten fünf Termine im Studienzentrum wahrzunehmen. Danach finden die Termine etwa alle 6 Monate statt und können oft mit normalen Praxis-/Klinikbesuchen kombiniert werden.

Eine Studienvisite dauert ca. eine Stunde.

- Insgesamt wird die Studie für Sie ca. 48 Monate dauern, dies ist jedoch abhängig davon, wie schnell die Teilnehmer in die Studie eingeschlossen werden können.
- Bei jedem Studienbesuch werden Sie zu Ihrer Gesundheit und Medikamenteneinnahme befragt. Es werden Ihnen außerdem etwa 20 ml Blut für routinemäßige Bluttests abgenommen (entspricht einer ungefähren Menge von 2 Esslöffeln). Zu Beginn der Behandlung, nach 12 Wochen und bei der Abschlussuntersuchung werden weitere 20 ml Blut für weitere Tests entnommen. Zusätzlich werden Sie um eine Urinprobe gebeten.
- Zu Beginn der Einnahme der Studienmedikation, nach 6 Monaten und danach einmal im Jahr werden Sie gebeten, zwei Fragebögen zu Ihrer Lebensqualität auszufüllen. Dies wird etwa 10 Minuten Zeit in Anspruch nehmen.
- Diese Studie wurde von Wissenschaftlern am Universitätsklinikum in Groningen, Niederlande, entwickelt und wird von dort aus koordiniert. Insgesamt sollen 1500 Personen daran teilnehmen.
- Der Arzneimittelhersteller AstraZeneca finanziert die Studie und stellt die Medikamente zur Verfügung.
- Nachfolgend erhalten Sie nähere Informationen über die Studie. Bitte lesen Sie die Informationen sorgfältig durch und wenden Sie sich mit Ihren Fragen an den Prüfer/die Prüferin, der/die Ihnen diese Patienteninformation ausgehändigt hat.
- Darüber hinaus können sie mit ihrem Partner/Ihrer Partnerin, ihrer Familie oder ihren Freunden über diese Studie sprechen. Im Anschluss können Sie Ihre schriftliche Einwilligung zur Teilnahme geben, wenn Sie sich zur Teilnahme entschieden haben.

Obwohl aus Gründen der besseren Lesbarkeit zur Bezeichnung von Personen die maskuline Form gewählt wurde, beziehen sich die Angaben selbstverständlich auf Angehörige beider Geschlechter.

# Patienteninformation

Sehr geehrte Patientin, sehr geehrter Patient, wir möchten Sie einladen, an der nachfolgend beschriebenen klinischen Prüfung teilzunehmen.

Was ist die Schlüsselfrage, die mithilfe der Studie beantwortet werden soll?

Das Universitätsklinikum Groningen (UMCG) in den Niederlanden hat diese Studie ins Leben gerufen, um die Wirksamkeit des Medikaments Dapagliflozin (Forxiga®) in der Prävention von Nieren- und Herzversagen sowie dessen Verträglichkeit und Sicherheit zu untersuchen. Hierfür wird der Effekt von Dapagliflozin mit dem Effekt eines Scheinmedikaments ohne Wirkstoff (ein so genanntes Placebo) miteinander verglichen.

Welche Wirkungsweise hat das untersuchte Medikament?

Das in dieser Studie verwendete Medikament Dapagliflozin (Forxiga®), gehört zur einer neuen Art von Medikamenten, sogenannten SGLT2-Hemmern, die seit einigen Jahren zur Behandlung von Diabetes, Herz-Kreislauf-Erkrankungen und Erkrankungen der Nieren zugelassen sind. Mehrere große Studien haben die positiven Effekte der SGLT2-Hemmer bestätigt, einschließlich der Reduktion von Eiweiß (Protein) im Urin und dem Schutz vor Nieren- und Herzversagen. Zusätzlich wurden SGLT2-Hemmer als gut verträglich und sicher eingestuft.

An den bisher durchgeführten Studien waren jedoch Patienten mit schlechter Nierenfunktion (eGFR <25 ml/min/1,73 m2, Dialysepatienten und Empfänger von Nieren-Transplantaten) nicht beteiligt. Dies ist bedauerlich, da gerade bei diesen Patienten ein erhöhtes Risiko für terminale Niereninsuffizienz, Herzinsuffizienz oder Tod besteht.

Tierversuchen und retrospektive Analysen großer klinischer Studien mit SGLT2-Hemmer haben gezeigt, dass auch diese Gruppe von Patienten mit schweren Nierenerkrankungen von einer Behandlung mit SGLT2-Hemmern profitieren können. Daher soll in dieser Studie die schützende Wirkung von Dapagliflozin vor Herz- und Nierenversagen bei Patienten mit schwerer Nierenerkrankung untersucht werden.

Muss ich an der Studie teilnehmen?

Nein, Sie müssen nicht an dieser Studie teilnehmen. Die Teilnahme ist freiwillig. Ihre Teilnahme an dieser Studie hat keinen Einfluss auf eine andere medizinische Behandlung, die Sie möglicherweise brauchen oder von Ihrem behandelnden Arzt erhalten.

Bitte lesen Sie diese Information über die Studie sorgfältig durch, bevor Sie eine Entscheidung über Ihre Teilnahme treffen. Zögern Sie nicht, vor Ihrer Zustimmung Ihre Fragen an das Studienteam zu richten. Sprechen Sie mit Ihrer Familie oder Ihrem Hausarzt, wenn Sie diese in die Entscheidung einbeziehen möchten. Sollten Sie sich für eine Teilnahme entscheiden, wird Ihnen bei Ihrem ersten Besuch im Studienzentrum ein Studienarzt die Studie erklären und Ihnen Gelegenheit geben, weitere Fragen zu stellen.

Wie ist der Ablauf der Studie?

Insgesamt wird Ihre Teilnahme an der Studie etwa 48 Monate dauern. Die tatsächliche Dauer ist jedoch abhängig vom Erreichen der erforderlichen Gesamtzahl an Teilnehmern. In den ersten 6 Monate finden 5 Studienvisiten im Studienzentrum statt. Danach finden die Besuche alle sechs Monate statt und können oft mit normalen Klinikbesuchen kombiniert werden. Ein Studienbesuch wird etwa eine Stunde dauern.

Was geschieht im Rahmen des ersten Studientermins?

Im Rahmen Ihres ersten Besuchs, der sogenannten Screening-Visite, wird der Studienarzt Sie über die Studie aufklären, Ihre Fragen beantworten und Sie bitten Ihre Zustimmung zur Teilnahme zu geben. Nachdem Sie Ihre schriftliche Einwilligung gegeben haben, werden wir überprüfen, ob Sie für die Teilnahme an der Studie geeignet sind. Dazu werden folgende Untersuchungen durchgeführt:

- Eine routinemäßige körperliche Untersuchung. Der Prüfarzt wird zum Beispiel Ihr Herz und Ihre Lungen abhören sowie Ihren Blutdruck und Ihre Herzfrequenz messen.
- Außerdem wird der Prüfarzt Ihr Alter, ethnische Zugehörigkeit, Krankengeschichte und die Medikamente, die Sie derzeit einnehmen, erfassen sowie Ihren Alkohol- und Tabakkonsum dokumentieren.
- Eine Blutabnahme, um u.a. Ihre (Rest-)Nierenfunktion zu bestimmen.
- Bei Frauen im gebärfähigen Alter wird ein Schwangerschaftstest durchgeführt.

Möglicherweise benötigen wir weitere Informationen von Ihrem Hausarzt und/oder Ihrem behandelnden Facharzt, um Fragen zu Ihrer Gesundheit zu klären. Dies bezieht sich z.B. auf Unklarheiten bezüglich Ihrer Medikamenteneinnahme sowie auf vergangenen Operationen und/oder Behandlungsmethoden.

Was geschieht im Rahmen der weiteren Studientermine?

Wenn Sie für die Teilnahme an der Studie in Frage kommen, werden Sie beim Randomisierungsbesuch einer Behandlungsgruppe zugeteilt. Im Anschluss an diesen Termin wird Ihnen das Studienmedikament ausgegeben.

Entsprechend Ihrer Zuteilung erhalten Sie entweder Dapagliflozin 10 mg (1 Tablette/Tag) oder Placebo (1 Tablette/Tag) für die gesamte Dauer der Studie. Das Arzneimittel sollte einmal täglich morgens (zwischen 7 und 9 Uhr) mit etwas Wasser eingenommen werden. Dabei spielt es keine Rolle, ob Sie das Arzneimittel vor oder nach dem Frühstück einnehmen.

Welche Behandlung Sie erhalten, wird von einem Computer nach dem Zufallsprinzip (ähnlich dem Werfen einer Münze) entschieden. Dieser Vorgang wird als Randomisierung bezeichnet. Weder Sie noch das Studienteam am Prüfzentrum werden wissen, welche Behandlung (Dapagliflozin oder Placebo) Sie erhalten. Dadurch soll sichergestellt werden, dass die aus der Studie gewonnenen

Ergebnisse zuverlässig und glaubwürdig sind. Für wichtige Fragen betreffend Ihres Gesundheitszustandes kann die Information über die Behandlung jedoch eingeholt werden.

Nachdem Sie mit der Einnahme des Studienmedikaments begonnen haben, findet nach 2 bis 3

Wochen eine erste Sicherheitsuntersuchung statt. 12 und 26 Wochen sowie 6 Monate nach

Studieneinschluss finden weitere Studienvisiten statt. Anschließend finden die Studientermine immer im Abstand von 6 Monaten statt. Diese werden, wenn möglich, mit Ihren regulären Kontrollbesuchen kombiniert.

Zu Beginn der Einnahme der Studienmedikation (Randomisierungsbesuch), nach 6 Monaten und danach einmal im Jahr werden sie gebeten, jeweils zwei Fragebögen betreffend Ihrer Lebensqualität auszufüllen. Dies wird etwa 10 Minuten Zeit in Anspruch nehmen.

Die Studienmedikation und die zusätzlichen Messungen im Rahmen der Studie sind unabhängig von einer möglichen Behandlung, die Sie im Rahmen Ihrer medizinischen Standardversorgung erhalten. In Anhang A finden Sie eine Übersicht der einzelnen Maßnahmen während der jeweiligen Studienvisite.

Blut- und Urinproben

Bei jedem Besuch werden Ihnen etwa 20 ml Blut für routinemäßige Bluttests abgenommen. Diese Menge entspricht etwa zwei Esslöffeln. Zu Beginn der Behandlung (beim Randomisierungsbesuch), nach 12 Wochen und bei der Abschlussuntersuchung werden Ihnen zusätzliche 20 ml Blut entnommen, womit nach Abschluss der Studie weitere Tests durchgeführt werden können.

Bei der Screening-Visite, zu Beginn der Behandlung und danach alle 6 Monate werden Sie gebeten, eine Urinprobe am Morgen des Studienbesuchs abzunehmen. Um bei Dialysepatienten die Restnierenfunktion und die Effizienz der Dialyse zu beurteilen, sollten sie zu Beginn der Behandlung (Randomisierung) und danach alle 6 Monate statt des Morgenurins eine Probe des 24-Stunden-Sammelurins abgeben.

Zur Erfassung Ihres Gesundheitszustandes wird beim Screeningbesuch vom Prüfarzt oder dem Studienpersonal eine körperliche Routineuntersuchung durchgeführt (z.B. Blutdruck, Herzfrequenz und Gewichtsmessung). Bei Frauen im gebärfähigen Alter wird zusätzlich ein Schwangerschaftstest durchgeführt. Darüber hinaus werden Sie bei jedem Besuch über Ihre Medikamenteneinnahme, sowie über mögliche Ereignisse und unerwünschte Wirkungen der Studienmedikation befragt. Dazu zählen Blaseninfektionen, Infektionen der Genitalien und niedriger Blutzucker. Falls Sie dialysepflichtig geworden sind und/oder wegen einer Herzinsuffizienz im Krankenhaus waren, wird dies ebenfalls erfasst.

Was sollte ich bei einer Studienteilnahme beachten?

Wir möchten, dass die Ergebnisse dieser Studie verlässlich sind, daher möchten wir mit Ihnen folgende Vereinbarungen treffen:

- Es ist wichtig, dass Sie Ihr Studienmedikament nach Anweisung des Studienpersonals täglich zuverlässig einnehmen.
- Bitte teilen Sie Ihrem Studienarzt mit, ob Sie im vergangenen Jahr an einer anderen Forschungsstudie teilgenommen haben oder derzeit an einer anderen Forschungsstudie teilnehmen. Die Teilnahme an weiteren Studien sollte vorher mit dem Studienarzt besprochen werden, um Sie vor möglichen Schäden z. B. durch die Entnahme zusätzlicher Blutproben, mögliche Wechselwirkungen von Medikamenten oder anderen Gefahren zu schützen.
- Bitte befolgen Sie die Anweisungen des Studienpersonals und nehmen Sie alle geplanten Studienbesuchen wahr.
- Bitte tragen Sie den Teilnehmerausweis bei sich, zum Beispiel in Ihrer Brieftasche. Darauf ist vermerkt, dass Sie an dieser Studie teilnehmen und wen Sie oder Ihr behandelndes Fachpersonal im Notfall benachrichtigen müssen. Zeigen Sie diese Karte vor, wenn Sie einen (anderen) Arzt aufsuchen.
- Bitte bringen Sie Ihre unbenutzten Studienmedikamente und alle leeren Behältnisse zu jedem Ihrer Studienbesuche mit und geben Sie bitte Bescheid, wenn Studienmedikamente verloren gehen oder fehlen.
- In den folgenden Situationen wenden Sie sich bitte an den Prüfarzt:
  - Sie möchten andere Medikamente einnehmen, auch wenn es sich um homöopathische Mittel, Naturheilmittel, Vitamine oder nicht verschreibungspflichtige Medikamente handelt
  - Sie werden ins Krankenhaus eingeliefert oder dort behandelt o Sie fühlen sich plötzlich unwohl o Sie möchten nicht mehr an der Studie teilnehmen
  - Ihre Telefonnummer, Adresse oder E-Mail-Adresse hat sich geändert

Welche Vorteile bringt eine Studienteilnahme?

Von Ihrer Teilnahme an der Studie haben Sie möglicherweise keinen persönlichen Nutzen. Wenn Sie das Prüfpräparat erhalten, können Sie aber von möglichen positiven Effekten der Behandlung profitieren. Es wird erwartet, dass sich Dapagliflozin positiv auf den Blutdruck und die Eiweißausscheidung im Urin auswirken kann, sowie Nieren- und/oder Herzversagen vorbeugen und vor Todesfällen schützen kann. Da dies noch nicht erwiesen ist, ist es jedoch möglich, dass Sie durch Ihre Teilnahme nicht den erhofften Nutzen haben. Einen zusätzlichen Nutzen durch die Teilnahme haben Sie auch dann nicht, wenn Sie statt des Prüfpräparat das Placebo erhalten.

Durch Ihre Studienteilnahme leisten Sie allerdings einen wichtigen Beitrag zur Gewinnung von neuen Erkenntnissen und unterstützen die Forscher dabei, möglicherweise die Behandlung von Herz- und Nierenerkrankungen zu verbessern.

Welche Risiken sind mit einer Teilnahme verbunden?

Die meisten Medikamente haben Nebenwirkungen, die bei einigen Personen auftreten, während andere Personen unter keinerlei Nebenwirkungen leiden.

Bei der Einnahme von Dapagliflozin wurden bei den bisher untersuchten Patientengruppen folgende Nebenwirkungen beobachtet (mehr dazu finden Sie in Anhang B):

- Zunahme der Urinmenge und/oder des Durstgefühls
- Blasenentzündung
- Infektionen der Vagina oder des Penis und/oder der Harnröhre

Da Dapagliflozin-Tabletten Laktose enthalten, kann es bei Laktoseintoleranz zu Beschwerden kommen.

Durch die Einnahme von Dapagliflozin können jedoch auch Nebenwirkungen auftreten, über die wir noch keine Kenntnisse besitzen. Da die Anwendung von Prüfpräparaten immer mit Risiken verbunden ist, wird Ihr Gesundheitszustand engmaschig überwacht. Falls Nebenwirkungen auftreten, informieren Sie bitte umgehend den Prüfarzt, damit Sie bei Bedarf angemessen behandelt werden können. Die Meldung von Nebenwirkungen dient Ihrem eigenen Schutz. Weitere Informationen über Dapagliflozin finden Sie in der Packungsbeilage.

Die Blutentnahme ist für Sie mit den geringen Risiken einer normalen Blutentnahme verbunden, das heißt, an der Einstichstelle können Schmerzen, lokaler Reizung, Blutungen oder ein Bluterguss (blauer Fleck) entstehen. Es besteht ein geringes Risiko für Benommenheit und/oder Ohnmacht. In seltenen Fällen kann sich die Einstichstelle zudem entzünden oder Nerven können geschädigt werden, was zu anhaltenden Missempfindungen, vermindertem Tastempfinden und anhaltenden Schmerzen führen kann. Um diese Risiken zu minimieren, werden wir die Blutentnahmen so weit wie möglich mit den Blutentnahmen kombinieren, die Teil der regulären Behandlung sind.

Welche Nachteile hat eine Studienteilnahme?

Die Teilnahme an der Studie kann einige Nachteile haben, die im Folgenden aufgeführt sind:

- Sie können die bereits beschriebenen Nebenwirkungen von Dapagliflozin verspüren.
- Die Teilnahme an der Studie wird Sie zusätzliche Zeit kosten.
- Sie sollten sich an die Vereinbarungen im Rahmen des Studienablaufs halten.

Es besteht die Möglichkeit, dass wir während dieser Studie Erkenntnisse gewinnen, die nicht direkt für die Untersuchung relevant sind, aber für Ihre Gesundheit oder die Ihrer Familienangehörigen wichtig sind. In diesem Fall wird Ihr Hausarzt oder Facharzt mit Ihnen besprechen, wie Sie damit umgehen sollen. Die Kosten hierfür werden von Ihrer Krankenkasse übernommen.

Informationen zur Schwangerschaftskontrolle

Wenn Sie schwanger sind, eine Schwangerschaft planen oder einen Säugling stillen, können Sie nicht an dieser Studie teilnehmen. Sie dürfen während der Studie bis zu 4 Wochen nach der letzten Einnahme des Studienmedikaments nicht schwanger werden. Es wurden bislang keine Studien mit Dapagliflozin bei schwangeren oder stillenden Frauen, durchgeführt. Daher ist nicht bekannt, ob Dapagliflozin für ungeborene oder gestillte Kinder sicher ist. Bei Frauen, die schwanger werden können, führen wir Schwangerschaftstests durch. Weiterhin sollten sich Frauen im gebärfähigen Alter mit der Anwendung einer hochwirksamen Empfängnisverhütungsmethode während der gesamten Studie und für eine Woche nach Studienende einverstanden erklären (hochwirksame Empfängnisverhütungsmethoden werden in der Fußnote am Seitenende genannt^^[[1]](#footnote-1)^^).

Falls Sie während der Studie schwanger werden, teilen Sie dies dem Studienteam bitte sofort mit.

Sie sollten die Studie dann so schnell wie möglich in Absprache mit dem Studienarzt beenden.

__________________________________

Wann ist die Studie beendet?

Während der Studie werden Sie über Änderungen der Studienabläufe, neu entdeckte Nebenwirkungen oder wichtige Erkenntnisse informiert, die sich auf Ihre Gesundheit oder Ihre Bereitschaft zur Teilnahme auswirken könnten. Das Studienteam wird Sie fragen, ob Sie weiterhin bereit sind, an der Studie teilzunehmen.

In den folgenden Fällen wird die Studie für Sie beendet:

- Alle Studienbesuche und Untersuchungen wurden gemäß Zeitplan abgeschlossen.
- Sie sind schwanger geworden.
- Sie wollen selbst nicht mehr an der Studie teilnehmen. Das ist jederzeit möglich. Teilen Sie dies bitte dem Studienteam mit. Sie müssen nicht angeben, warum Sie die Teilnahme beenden möchten. Dennoch werden Sie zu einer Nachuntersuchung eingeladen.
- Der Prüfer/die Prüferin ist der Meinung, dass es besser für Sie wäre, die Teilnahme zu beenden.

Auch in diesem Fall werden Sie zu einer Nachuntersuchung eingeladen.

- Eine der folgenden Behörden entscheidet, dass die Studie beendet werden soll: o der Auftraggeber der Studie o eine Behörde

o die medizinische Ethik-Kommission, welche die Studie bewertet

Was geschieht, wenn ich nicht länger an der Studie teilnehmen möchte?

Ihre Teilnahme an der Studie ist freiwillig. Sie können jederzeit Ihre Einwilligung ohne Angabe von Gründen und ohne Nachteile widerrufen. Im Fall eines solchen Widerrufs werden keine weiteren Daten über Sie erhoben. Die bis zu diesem Zeitpunkt bereits erhobenen Daten und die gesammelten Blut- und Urinproben werden weiterhin aufbewahrt und verwendet, soweit dies erforderlich ist, um die Wirkung des Studienmedikaments festzustellen. Außerdem muss sichergestellt sein, dass Ihre schutzwürdigen Interessen nicht beeinträchtigt werden und/oder die Pflicht zur Vorlage vollständiger Zulassungsunterlagen erfüllt wird. Bei einem Widerruf Ihrer Einwilligung wird das gesammelte Körpermaterial grundsätzlich vernichtet. Für eine weitere Verwendung der Proben würden wir Ihre Einwilligung über eine separate Einwilligungserklärung einholen.

Sollten Sie Ihre Studienteilnahme vorzeitig beenden, hat das keinerlei Auswirkung auf Ihre reguläre Behandlung und Ihre Rechte als Patient.

Wenn Sie von einem Arzt gebeten werden, das Studienmedikament abzusetzen, oder Sie sich gegen die weitere Teilnahme entscheiden, wäre es hilfreich, wenn Sie dem Studienteam weitere Kontaktaufnahmen erlauben würden.

Was geschieht nach Abschluss der Studie?

Wenn Sie es wünschen, können Sie die Arzneimittel, die Sie während der Studie eingenommen haben, nach dem Ende der Studie weiterverwenden. Dies geschieht dann durch ärztliche Verschreibung durch Ihren behandelnden Arzt.

Etwa ein Jahr nach dem Ende Ihrer Teilnahme werden Sie von den Forschern über die wichtigsten Ergebnisse der Studie informiert. Das betreuende Studienteam kann Ihnen dann auch mitteilen, welche Behandlung Sie erhalten haben. Sollten Sie diese Information nicht wünschen, geben Sie bitte den Verantwortlichen Ihres Studienzentrums Bescheid.

Wer koordiniert diese Studie?

Diese Studie wurde von Wissenschaftlern des Universitätsklinikums Groningen (UMCG) entwickelt und wird von dort aus koordiniert. Insgesamt sollen 1500 Personen an der Studie teilnehmen, 250 davon in Deutschland. Die Durchführung einer solchen Studie erfordert ein hohes Maß an Zusammenarbeit zwischen Forschern verschiedener Kliniken und Dialysezentren und kann sehr kostspielig sein. Der Arzneimittelhersteller AstraZeneca finanziert die Studie in Deutschland und stellt die Medikamente zur Verfügung. Die zuständige Ethik-Kommission (Ethik-Kommission der Universität Würzburg) hatte keine ethischen und rechtlichen Einwände gegen die Durchführung dieser Studie.

Wie gehen wir mit Ihren Daten und Bioproben um?

Wenn Sie in die Studienteilnahme einwilligen, erklären Sie sich damit einverstanden, dass Ihre Daten und Bioproben (Blut und Urin) erfasst, verwendet und gespeichert werden. Ihre Teilnahme an dieser Studie wird auch in Ihrer Krankenakte in Ihrem Krankenhaus oder Dialysezentrum vermerkt. Dies geschieht zu Ihrer eigenen Sicherheit.

Welche Informationen werden über mich erfasst?

Während Ihrer Studienteilnahme werden Sie bei Ihren Studienterminen dem Studienpersonal gegenüber personenbezogene Daten (und in Form von Blut- und Urintestergebnissen, die von Ihnen im Krankenhaus aufbewahrt werden) über sich offenlegen. Dazu gehören Name, Geschlecht, Anschrift, Geburtsdatum, sowie Informationen über Ihren Gesundheitszustand und Ihre Krankengeschichte.

Daten, mit denen Sie direkt identifiziert werden können (z.B. Name, Kontaktinformationen, vollständiges Geburtsdatum), werden nur in Ihrem lokalen Studienzentrum gespeichert und nicht nach außen gegeben.

Warum erheben, verwenden und speichern wir Ihre Daten und Bioproben?

Wir erheben, verwenden und speichern Ihre Daten und Ihre Bioproben, um die Fragestellungen dieser Studie zu beantworten. Wir bitten Sie um Ihr Einverständnis für die Verwendung Ihrer Daten und Bioproben. Wenn Sie dies nicht wünschen, können Sie nicht an dieser Studie teilnehmen.

Wie schützen wir Ihre personenbezogenen Daten?

Um Ihre Privatsphäre zu schützen, werden die über Sie im Rahmen dieser Studie erfassten Informationen sowie die Ihnen entnommenen Proben „pseudonymisiert“. Pseudonymisiert bedeutet, dass Ihre Gesundheitsinformationen sowie die Blut- und Urinproben mit Identifikationsnummern gekennzeichnet werden, die ausschließlich über einen Computer und nicht über Ihren Namen mit Ihnen in Verbindung gebracht werden können. Ihre Daten, Urin- und Blutproben werden ausschließlich in pseudonymisierter Form verarbeitet und weitergegeben.

Alle Berichte, Veröffentlichungen oder Präsentationen, die aus dieser Studie hervorgehen könnten, werden keine Informationen enthalten, die Sie direkt identifizierbar machen.

Die Liste, die Ihren Code Ihrem Namen zuordnet, wird an einem sicheren Ort in Ihrem lokalen Studienzentrum aufbewahrt.

Es lässt sich niemals völlig ausschließen, dass es jemandem gelingt, anhand der codierten Daten Rückschlüsse auf Ihre Person zu ziehen. Allerdings ist das Risiko einer unerlaubten Feststellung Ihrer Identität sehr gering.

Wer kann Ihre Daten einsehen?

Einige Personen haben Zugang zu Ihren persönlichen Daten. Sie überprüfen, ob die Forscher die Studie ordnungsgemäß und zuverlässig durchführen. Die folgenden Personen/Institutionen können Zugang zu Ihren Daten erhalten:

- Die Mitglieder des Ausschusses, der die Sicherheit der Studie überwacht.
- Ein vom Sponsor beauftragter Prüfer (z.B. Auditoren und Monitore).
- Nationale und internationale Aufsichtsbehörden.

Diese Personen werden Ihre Daten vertraulich behandeln. Eine Weitergabe Ihrer Daten erfolgt in diesem Zusammenhang nicht.

Ihre pseudonymisierten Daten werden zur Analyse der Studienergebnisse verwendet. Es könnte außerdem erforderlich sein, die pseudonymisierten Daten an Behörden weiterzugeben oder mit anderen Wissenschaftlern zu teilen.

Während und nach dem Ende dieser Studie können wir Ihre pseudonymisierten Daten und Bioproben auch in Ländern außerhalb der Europäischen Union senden, um die Daten zu speichern und Blut und Urin zu analysieren, damit wir besser verstehen, wie Dapagliflozin wirkt. Diese Länder haben möglicherweise ein niedrigeres Datenschutzniveau als die EU. Es liegt kein Angemessenheitsbeschluss der Europäischen Kommission vor und es können keine behördlich genehmigten Datenschutzklauseln angewendet werden. Die Universität Groningen sichert zu, auch in diesen Fällen für eine vertragliche Verpflichtung der Forschungspartner zur Einhaltung des EU-Datenschutz-Niveaus zu sorgen, soweit dies rechtlich möglich ist. Dennoch besteht das Risiko, dass staatliche oder private Stellen auf Ihre Daten zugreifen, obwohl dies nach dem europäischen Datenschutzrecht nicht zulässig wäre. Beispielsweise kann in den USA ein Zugriff durch Geheimdienste auch ohne richterlichen Beschluss erfolgen. Zudem kann es sein, dass Ihnen dort weniger oder schlechter durchsetzbare Betroffenenrechte zustehen und es keine unabhängige Aufsichtsbehörde gibt, die Sie bei der Wahrnehmung Ihrer Rechte unterstützen könnte. Eine Weitergabe der von Ihnen erhobenen Daten (und gewonnenen Bioproben) kann in diesem Fall nur erfolgen, wenn Sie dem ausdrücklich zugestimmt haben (s. optionale Aspekte Einwilligungserklärung).

Wie lange speichern wir Ihre Daten?

Wie gesetzlich vorgeschrieben, speichern wir Ihre Daten 25 Jahre lang. Sie werden so lange aufbewahrt, um neue Bewertungen im Zusammenhang mit dieser Forschung vornehmen zu können. Sobald dies nicht mehr erforderlich ist, werden wir Ihre Datenlöschen.

Dürfen wir Ihre Daten und Bioproben für andere Forschungszwecke verwenden?

Ihre Daten und Bioproben können auch nach Abschluss dieser Studie für andere wissenschaftliche Forschungen auf dem Gebiet der Nieren- und Herzinsuffizienz von Bedeutung sein. Zu diesem Zweck möchten wir Ihre Daten und Bioproben für 25 Jahre aufbewahren. In der Einwilligungserklärung geben Sie an, ob Sie mit der Nutzung Ihrer Daten für andere Forschungsfragen einverstanden sind.

Können Sie Ihre Zustimmung zur Verwendung Ihrer Daten widerrufen?

Sie können Ihre Zustimmung zur Verwendung Ihrer Daten jederzeit widerrufen. Dies gilt sowohl für die Verwendung in dieser Studie als auch für die Verwendung in anderen Studien. Wenn Sie Ihre Studienteilnahme widerrufen werden Ihre Daten gelöscht. Eine Weiterverwendung von Daten ist nur im Rahmen der gesetzlichen Ausnahmeregeln möglich.

Welche Datenschutzrechte habe ich?

1. Recht auf Auskunft, Art. 15 DSGVO

Sie haben das Recht auf Auskunft über die Sie betreffenden, gespeicherten, personenbezogenen Daten.

1. Recht auf Berichtigung, Art. 16 DSGVO

Wenn Sie feststellen, dass unrichtige Daten zu Ihrer Person verarbeitet werden, können Sie Berichtigung verlangen. Unvollständige Daten müssen unter Berücksichtigung des Zwecks der Verarbeitung vervollständigt werden.

1. Recht auf Löschung, Art. 17 DSGVO

Sie haben das Recht, die Löschung Ihrer Daten zu verlangen, wenn bestimmte Löschgründe vorliegen. Dies ist insbesondere der Fall, wenn diese zu dem Zweck, zu dem sie ursprünglich erhoben oder verarbeitet wurden, nicht mehr erforderlich sind oder Sie Ihre Einwilligung widerrufen.

1. Recht auf Einschränkung der Verarbeitung, Art. 18 DSGVO

Sie haben das Recht auf Einschränkung der Verarbeitung Ihrer Daten. Dies bedeutet, dass Ihre Daten zwar nicht gelöscht, aber gekennzeichnet werden, um ihre weitere Verarbeitung oder Nutzung einzuschränken.

1. Recht auf Datenübertragbarkeit, Art. 20 DSGVO

Sie haben das Recht, die Daten, die Sie uns zur Verfügung gestellt haben, in einem gängigen elektronischen Format von uns zu verlangen.

1. Recht auf Widerspruch gegen unzumutbare Datenverarbeitung, Art. 21 DSGVO

Sie haben grundsätzlich ein allgemeines Widerspruchsrecht auch gegen rechtmäßige Datenverarbeitungen, die im öffentlichen Interesse liegen, in Ausübung öffentlicher Gewalt oder aufgrund des berechtigten Interesses einer Stelle erfolgen.

Möchten Sie Ihre Datenschutzrechte wahrnehmen?

- Wollen Sie von einem oder mehreren der genannten Rechten Gebrauch machen, kontaktieren Sie bitte Ihren Studienarzt. Bei Anliegen zur Datenverarbeitung und zur Einhaltung der datenschutzrechtlichen Anforderungen können Sie sich auch an folgende Datenschutzbeauftragte wenden:
- Verantwortlicher für die Verarbeitung Ihrer personenbezogenen Daten am Studienzentrum: [Kontaktdaten Prüfarzt einfügen]

- Der Datenschutzbeauftrage Ihres Studienzentrums ist:

[Kontaktdaten einfügen]

- Der Datenschutzbeauftragte des Sponsors, UMC Groningen, ist über privacy@umcg.nl zu erreichen.

- Wenn Sie Beschwerden über die Verarbeitung Ihrer personenbezogenen Daten haben, empfehlen wir Ihnen, diese zunächst mit dem Studienteam zu besprechen. Nur diesen ist aufgrund der Codierung Ihre Identität bekannt.
- Sie haben außerdem ein Beschwerderecht bei einer Datenschutzaufsichtsbehörde. Sollten Sie Bedenken hinsichtlich des Umgangs mit Ihren personenbezogenen Daten haben, können Sie sich an folgende Stellen wenden:
- Datenschutzaufsichtsbehörde des Bundeslandes, in dem Ihr Studienzentrum liegt: [Kontaktdaten einfügen]

- Für den Sponsor bzw. dessen Vertreter innerhalb der EU zuständige

Datenschutzaufsichtsbehörde:

Autoriteit Persoonsgegevens

PO Box 93374

2509 AJ DEN HAAG

Telefon: + 31-70-88 88 500

E-Mail: internationaal@autoriteitpersoonsgegevens.nl Homepage: https://autoriteitpersoonsgegevens.nl/nl

Werden Sie eine Vergütung für die Teilnahme an der Studie erhalten?

Das Studienmedikament sowie zusätzliche Tests und Messungen im Rahmen der Studie sind für

Sie kostenlos. Sie erhalten keine Aufwandsentschädigung, wenn Sie an dieser Studie teilnehmen.

Allerdings erhalten Sie eine Pauschale von 25 Euro für Reisekosten, wenn Sie Ihr Krankenhaus/ Dialysezentrum für zusätzliche Besuche außerhalb der regulären Versorgung aufsuchen.

Versicherung bei Studienteilnahme

Für alle Teilnehmer an dieser Studie wurde eine Versicherung abgeschlossen. Die Versicherung kommt für Schäden auf, die durch die Studie verursacht werden. Allerdings sind dadurch nicht alle möglichen Schäden abgedeckt: Eine Kopie der Versicherungsbedingungen erhalten Sie zusammen mit dieser Patienteninformation.

Wenn Sie vermuten, dass durch die Teilnahme an der klinischen Studie Ihre Gesundheit geschädigt oder bestehende Leiden verstärkt wurden, wird empfohlen dies unverzüglich dem

Versicherer

HDI-Global SE

HDI-Platz 1

30659 Hannover

Tel.: +49 (511) 645-0 Fax: +49 (511) 645-4545 zur Policen-Nr.: 57 010329 03013/03315 anzeigen, um Ihren Versicherungsschutz nicht zu gefährden.

Wir informieren Ihren Hausarzt und/oder Facharzt

Das Studienteam wird Ihrem Haus- und/oder Facharzt einen Brief/eine E-Mail schicken, um ihn/sie über Ihre Teilnahme an dieser Studie zu informieren. Dies dient Ihrer eigenen Sicherheit. Ihr Hausarzt und ggf. Ihr Facharzt werden über eventuelle Nebenbefunde informiert. Wenn Sie damit nicht einverstanden sind, können Sie nicht an dieser Studie teilnehmen.

Es ist möglich, dass wir während der Studie auf etwas stoßen, das für Ihre Gesundheit oder die Gesundheit Ihrer Familienmitglieder wichtig ist. Im Falle unerwarteter Befunde würde sich das Studienteam mit Ihrem Hausarzt in Verbindung setzen. Sie können mit Ihrem Hausarzt oder Facharzt besprechen, was in diesem Fall zu tun ist. Mit der Einwilligungserklärung erteilen Sie die Erlaubnis, Ihren Haus- oder Facharzt zu informieren.

Haben Sie noch Fragen?

Bei Fragen zur Studie wenden Sie sich bitte an das Studienteam in Ihrem Prüfzentrum. Wenn Sie mit anderen Aspekten der Studie unzufrieden sind und eine formelle Beschwerde einbringen möchten, können Sie dies bei der Studienzentrale in Würzburg tun.

Es existiert außerdem eine Kontaktstelle bei der zuständigen Bundesoberbehörde. Teilnehmer an klinischen Studien, ihre gesetzlichen Vertreter oder Bevollmächtigten können sich an diese Kontaktstelle wenden:

Bundesinstitut für Arzneimittel und Medizinprodukte

Fachgebiet Klinische Prüfung / Inspektionen

Kurt-Georg-Kiesinger-Allee 3, 53175 Bonn

Telefon: 0228 / 207-4318 Fax: 0228 / 207-4355

E-Mail: klinpruefung@bfarm.de

Vielen Dank, dass Sie diese Patienteninformation gelesen haben!

Unser Ziel ist es, Ihre Teilnahme an der Studie möglichst lohnenswert zu gestalten, während Sie uns und anderen dabei helfen, die Therapie für Personen, die unter einer schweren Nierenerkrankung leiden zu verbessern.

Bei Fragen wenden Sie sich bitte an die Studienzentrale:

Clinical Trial Office - Studienzentrale Deutschland

am Universitätsklinikum Würzburg (Deutsches Zentrum für Herzinsuffizienz)

Am Schwarzenberg 15, Haus A15

97078 Würzburg

Tel.: ++49 931 – 201 46373

Fax: ++49 931 – 201 646343

E-mail: ClinicalTrialOffice@ukw.de

Anhänge zu diesem Informationsschreiben

1. Überblick über die Studienbesuche und -verfahren
2. Mögliche Nebenwirkungen von Dapagliflozin
3. Einwilligungserklärung

Anhang A: Überblick über die Studienbesuche und -verfahren

|  | Screening |  | Behandlungsperiode | | | | (vorzeitige)  Beendigung |
| --- | --- | --- | --- | --- | --- | --- | --- |
| Besuche | 1 | 2 | 3 | 4 | 5 | 6, 7, 8,  9, 10,  11, 12… |  |
| Woche | -2 | 0 | 2 | 12 | 26 | Alle 6  Monate |  |
| Einwilligung nach Aufklärung | x |  |  |  |  |  |  |
| Randomisierung |  | x |  |  |  |  |  |
| Ausfüllen von Fragebögen |  | x |  |  | x | x* | x |
| Überprüfung der  Krankengeschichte | x |  |  |  |  |  | x |
| Überprüfung der  Medikamenteneinnahme | x | x | x | x | x | x | x |
| Erhebung von Vitalwerten | x | x | x | x | x | x | x |
| Körperliche Untersuchung | x | x** | x** | x** | x** | x** | x** |
| Schwangerschaftstest | x |  |  |  |  |  |  |
| Blutabnahme | x | x | x | x | x | x | x |
| Sammlung von Morgenurin | x | x |  | x | x | x | x |
| 24-Stunden-Urinsammlung  Nur Dialysepatienten |  | x |  | x | x | x | x |
| Aufbewahrung von Blut- und  Urinproben |  | x |  | x |  |  | x |
| Ausgabe der Studienmedikation |  | x | x | x | x | x |  |
| Überprüfung der  Studienmedikation |  |  | x | x | x | x | x |
| Aufzeichnung unerwünschter  Ereignisse |  | x | x | x | x | x | x |
| Überprüfung der Einnahme anderer Medikamente |  |  | x | x | x | x | x |
| * einmal jährlich  ** falls notwendig |  |  |  | | | |  |

Anhang B: Mögliche Nebenwirkungen von Dapagliflozin

Dapagliflozin (Forxiga®) wird derzeit weltweit für die Behandlung von Diabetes, Herz-Kreislauf- und Nierenerkrankungen verschrieben. Nebenwirkungen, Risiken und mögliche Nachteile sind bekannt und sorgfältig dokumentiert. Dapagliflozin wurde bisher nicht bei Menschen mit schwerer Nierenfunktionsstörung (weniger als 25 ml/min/1,73m2, Dialyse- und Transplantationspatienten) untersucht. Bei Ihnen können keine, einige oder alle der unten aufgeführten Nebenwirkungen auftreten:

Häufig (1-10 %):

- - Erhöhung der Urinproduktion:

Dapagliflozin erhöht die Zuckermenge im Urin, wodurch sich die Urinproduktion erhöht. Symptome: Sie müssen häufiger urinieren und bekommen mehr Durst. Anzeichen für einen zu hohen Flüssigkeitsverlust sind niedriger Blutdruck und Schwindelgefühl.

- - Harnwegsinfektionen (HWIs):

Dapagliflozin kann das Risiko von Harnwegsinfektionen erhöhen.

Symptome: Beschwerden beim Urinieren, verstärkter Harndrang, erhöhte Häufigkeit des Urinierens oder Fieber.

- - Genitalinfektionen:

Dapagliflozin kann das Risiko einer Hefepilzinfektion der Vagina, der Vulva oder des Penis erhöhen. Diese Infektionen treten häufiger bei Frauen und bei Menschen mit einer früheren Infektion dieser Art auf.

Symptome: Schmerzen oder Juckreiz, Ausfluss oder Hautausschlag

Selten (< 0,1 %):

- - Hautausschlag und/oder Juckreiz, Überempfindlichkeit - Niedriger Blutdruck (Hypoglykämie) ist sehr selten:

Diese Nebenwirkung tritt nur in Ausnahmefällen bei Patienten mit Diabetes auf. Symptome: Übermäßiges Schwitzen, Herzklopfen, Zittern, Schwindel, verschwommenes Sehen, Unwohlsein (wie Müdigkeit, Schwäche) und Unterzuckerung.

- - Eine diabetische Ketoazidose (DKA) ist sehr selten:

Dapagliflozin kann das Risiko einer diabetischen Ketoazidose erhöhen. Diese

Nebenwirkung tritt nur in Ausnahmefällen bei Patienten mit Typ-2-Diabetes auf. Symptome: Übelkeit, Erbrechen, Bauchschmerzen, Unwohlsein (wie Müdigkeit, Schwäche), Kurzatmigkeit und hoher Blutzucker.

Wenn diese Symptome bei Ihnen auftreten, müssen Sie sich sofort mit uns in Verbindung setzen.

Anhang C: Einwilligungserklärung

# EINWILLIGUNGSERKLÄRUNG

NOTWENDIGE Aspekte der Renal LIFECYCLE Studie

1. Ich bestätige, dass ich die Patienteninformation der RENAL LIFECYCLE Studie gelesen habe (siehe Versionsnummer und Datum weiter unten). Ich hatte ausreichend Gelegenheit über diese Informationen nachzudenken und Fragen zu stellen. Alle Fragen wurden zu meiner Zufriedenheit beantwortet.

RENAL LIFECYCLE Patienteninformation und Einwilligungserklärung GER V1.1 20.12.2022

1. Mir ist bewusst, dass meine Teilnahme an der RENAL LIFECYCLE Studie freiwillig ist und ich jederzeit meine Einwilligung wiederrufen kann ohne Angabe von Gründen, ohne dass meine reguläre medizinische Behandlung oder meine Rechte davon beeinflusst werden.
2. Ich bin damit einverstanden, dass Blut- und Urinproben für studienbezogene Analysen gewonnen und gespeichert werden. Ich erteile dem Studienteam die Erlaubnis, meinen Hausarzt/ Facharzt über unerwartete Ergebnisse der Studie, die für meine Gesundheit wichtig sind, zu informieren.
3. Ich erkläre mich damit einverstanden, dass autorisierte und zur Verschwiegenheit verpflichtete Personen der RENAL LIFECYCLE Studienzentrale Deutschland, der Universität Groningen sowie der zuständigen in- und ausländischen Überwachungsbehörden in meine beim Prüfzentrum vorhandenen personenbezogenen Daten, insbesondere meine Gesundheitsdaten, Einsicht nehmen, soweit dies für die Überprüfung der ordnungsgemäßen Durchführung der Studie notwendig ist. Für diese Maßnahme entbinde ich den Prüfarzt von der ärztlichen Schweigepflicht.
4. Ich bin damit einverstanden, dass mein Hausarzt / Facharzt über meine Teilnahme an der RENAL LIFECYCLE Studie informiert wird. Ich erteile dem Studienteam die Erlaubnis, von meinem Hausarzt/ Facharzt oder anderen Behörden Informationen über meine Medikamenteneinnahme und/oder meine Krankengeschichte anzufordern, falls dies für die Studie erforderlich ist.
5. Ich erkläre mich damit einverstanden, dass meine persönlichen Daten im Rahmen dieser Studie aufgezeichnet, gespeichert, analysiert und transferiert werden gemäß den Angaben in der RENAL LIFECYCLE Patienteninformation (Versionsnummer und Datum siehe oben). Die Weitergabe der Daten erfolgt in pseudonymisierter Form.
6. Mir ist bewusst, dass ich während der Studie und bis 4 Wochen nach dem Ende der Einnahme der Studienmedikation nicht schwanger werden darf. Das Studienteam hat mit mir besprochen, wie ich am besten verhindern kann, schwanger zu werden.
7. Ich bin mit der Teilnahme an der RENAL LIFECYCLE Studie einverstanden.

OPTIONALE Aspekte der RENAL LIFECYCLE Studie

(Bitte kreuzen Sie im entsprechenden Feld Ja oder Nein an)

Ja Nein

|  |  |
| --- | --- |

1. Ich bin damit einverstanden, dass meine Daten gespeichert werden, um sie fürandere Forschungszwecke zu verwenden, wie im Informationsschreiben

Ja Nein

angegeben.

|  |  |
| --- | --- |

1. Ich bin damit einverstanden, dass (übrig gebliebene) Blut- und Urinproben für zukünftige weitere Forschungsvorhaben gelagert und verwendet werden, gemäß

den Angaben in der RENAL LIFECYCLE Patienteninformation.

|  |  |
| --- | --- |

1. Ich bin damit einverstanden, dass meine pseudonymisierten Daten und Bioproben auch in Ländern außerhalb der Europäischen Union transferiert werden

Ja Nein

Ja Nein

|  |  |
| --- | --- |

1. Ich bin damit einverstanden, dass mich das Studienteam nach Abschluss dieser Studie kontaktiert und fragt, ob ich an einer Folgestudie teilnehmen möchte.

Ja Nein

|  |  |
| --- | --- |

1. Ich erteile dem Studienteam die Erlaubnis, mir nach Abschluss der Studie mitzuteilen, welche Behandlung ich erhalten habe.

=

Ja Nein

|  |  |
| --- | --- |

1. Ich bin damit einverstanden, dass meine E-Mail-Adresse für das digitale Ausfüllen der Fragebögen verwendet werden darf.

Vor- und Nachname des Patienten/ der Patientin in Druckbuchstaben:

…………………………………………………………………………………………………….

Unterschrift: …………………………………………. Datum: …….. / …….. / ………...

_____________________________________________________________________________

Ich erkläre, dass ich diese/n Teilnehmer/in umfassend über die oben genannte Studie informiert habe.

Sollten während der Studie Informationen bekannt werden, die die Zustimmung des/r Studienteilnehmers/In beeinflussen könnten, werde ich ihn/sie rechtzeitig darüber informieren.

Vor- und Nachname des Studienarztes/ der Studienärztin in Druckbuchstaben:

…………………………………………………………………………………………………..…

Unterschrift: ……………………………………….. Datum: ……. / ……… / ………...

Der/Die Patient/In erhält die vollständige Patienteninformation sowie eine unterzeichnete Fassung der Einwilligungserklärung.

**C) Dutch version**

v. 5.0_4Aug2023

**Proefpersoneninformatie voor deelname**

**aan medisch wetenschappelijk onderzoek**

**De RENAL LIFECYCLE studie**

*Officiële titel:*

*Een placebo gecontroleerde studie om het effect van dapagliflozine*

*op nier- en hartfalen te onderzoeken bij nierpatiënten*

*Doel:*

*We onderzoeken de mogelijke positieve effecten van dapagliflozine*

*in het voorkomen van nier- en hartfalen*

Geachte heer/mevrouw,

Met deze informatiebrief willen we u vragen of u wilt meedoen aan medisch-wetenschappelijk onderzoek. Meedoen is vrijwillig. U krijgt deze brief omdat u een nierziekte heeft. U leest hier om wat voor onderzoek het gaat, wat het voor u betekent, en wat de voor- en nadelen zijn. Het is veel informatie. Wilt u de informatie doorlezen en beslissen of u wilt meedoen? Als u wilt meedoen, kunt u het formulier invullen dat u vindt in **bijlage E**.

**Stel uw vragen**

U kunt uw beslissing nemen met de informatie die u in deze informatiebrief vindt. Daarnaast raden we u aan om dit te doen:

- Stel vragen aan de onderzoeker die u deze informatie geeft.

- Praat met uw partner, familie of vrienden over dit onderzoek.

- Stel vragen aan de onafhankelijk deskundige, dr. P. van Dijk (diabetes arts in het UMC Groningen)

- Lees de informatie op [www.rijksoverheid.nl/mensenonderzoek](http://www.rijksoverheid.nl/mensenonderzoek).

**1. Algemene informatie**

Het Universitair Medisch Centrum (UMC) Groningen heeft dit onderzoek opgezet. Hieronder noemen we het UMCG steeds de ‘opdrachtgever’. Onderzoekers, dit kunnen artsen en onderzoeksverpleegkundigen zijn, voeren het onderzoek uit in verschillende ziekenhuizen en dialysecentra. Voor dit onderzoek zijn 1500 proefpersonen uit verschillende landen nodig. In Nederland zullen naar verwachting 1250 proefpersonen meedoen. Het farmaceutische bedrijf Astra Zeneca levert de medicijnen voor dit onderzoek en heeft verder geen rol in het onderzoek. De medisch-ethische toetsingscommissie van het UMC Groningen heeft dit onderzoek goedgekeurd.

**2. Wat is het doel van het onderzoek?**

In dit onderzoek bekijken we hoe goed het middel dapagliflozine (Forxiga^®^) werkt in het voorkomen van nier- en hartfalen. We onderzoeken ook de verdraagzaamheid en veiligheid van dit middel. We vergelijken de werking van dapagliflozine daarom met de werking van een placebo. Een placebo is een middel zonder werkzame stof, een ‘nepmiddel’.

1. **Wat is de achtergrond van het onderzoek?**

U bent gevraagd om deel te nemen aan dit onderzoek omdat u een nierziekte hebt. Sinds enige jaren wordt een nieuwe klasse geneesmiddelen gebruikt, zogenaamde SGLT2-remmers. Het onderzoeksgeneesmiddel dapagliflozine behoort ook tot deze klasse. Dit zijn medicijnen die worden aanbevolen voor de behandeling van diabetes, hart- en vaatziekten en nierziekten. Het gebruik van deze geneesmiddelen is goedgekeurd door de Europese (EMA) en Nederlandse (CBG) overheid. Eerdere grote onderzoeken hebben verschillende positieve effecten van SGLT2-remmers aangetoond, waaronder een daling in de hoeveelheid eiwit in urine en bescherming tegen het ontstaan van nier- en hartfalen. Daarnaast werden SGLT2-remmers goed verdragen en bleken deze middelen veilig.

In de onderzoeken die tot nu toe zijn uitgevoerd, werden patiënten met een slechte nierfunctie (een nierfunctie van minder dan 25 ml/min/1,73 m^2^, dialysepatiënten en niertransplantatie ontvangers) niet meegenomen. Dit is erg jammer, aangezien vooral deze patiënten een hoog risico lopen op het ontstaan van eindstadium nierfalen, hartfalen en overlijden. Er is steeds meer bewijs uit dierproefstudies en achteraf analyses van grote klinische onderzoeken dat SGLT2-remmers ook werkzaam kunnen zijn in het voorkomen van nierfalen, hartfalen en overlijden bij patiënten met ernstige nierziekte, waaronder dialyse en niertransplantatie patiënten. Daarom willen de onderzoekers nu onderzoeken of en hoe SGLT2-remmers werken op hart- en nierfalen bij patiënten met ernstige nierziekte, waaronder dialyse en niertransplantatiepatiënten.

**4. Hoe verloopt het onderzoek?**

*Hoelang duurt het onderzoek?*

Doet u mee met het onderzoek? Dan duurt dat in totaal ongeveer 48 maanden. Het onderzoek kan iets langer of korter duren dan de geplande 48 maanden. Dit hangt af van hoe snel het totale aantal deelnemers dat nodig is, is bereikt. De tijd die we van u vragen in het onderzoek is ongeveer 1 uur per bezoek. Bezoeken vinden gedurende het grootste deel van de studie eens per half jaar plaats. Deze bezoeken kunnen vaak gecombineerd worden met normale ziekenhuisbezoeken.

*Stap 1: bent u geschikt om mee te doen?*

Nadat u besloten heeft mee te willen doen aan dit onderzoek en het toestemmingsformulier heeft ondertekend, willen we eerst weten of u geschikt bent om mee te doen.

Daarom doen we een aantal onderzoeken:

- Lichamelijk onderzoek. De onderzoeker luistert bijvoorbeeld naar uw hart en longen en meet uw bloeddruk en hartslag.
- Daarnaast zal de onderzoeker uw leeftijd, afkomst, medische voorgeschiedenis en medicijnen die u momenteel gebruikt en uw alcohol of drugsgebruik registreren.
- Bloedafname om o.a. uw (rest) nierfunctie te bepalen.
- Bij vrouwen in de vruchtbare leeftijd wordt een zwangerschapstest uitgevoerd.

Het kan nodig zijn dat wij meer informatie nodig hebben van uw huisarts en/of behandelend medisch specialist. Dit zullen wij uiteraard alleen doen als het in verband is met uw eigen veiligheid. Voorbeelden van wanneer wij om meer informatie vragen zijn wanneer uw medicatiegebruik niet helemaal duidelijk is of wanneer wij meer informatie nodig hebben over operaties en/of behandelmethodes die u in het verleden heeft gehad. Uiteraard zullen wij u op de hoogte stellen voordat we contact opnemen met uw huisarts en/of behandelend medisch specialist.

*Stap 2: de behandeling*

Het onderzoek bestaat uit dapagliflozine 10 mg (1 tablet) of placebo (1 tablet) per dag gedurende ongeveer 48 maanden. Loting bepaalt of u dapagliflozine of placebo krijgt. U en de onderzoeker weten niet in welke groep u zit. Als het voor uw gezondheid belangrijk is, kan dit wel worden opgezocht.

De studie medicatie dient u 1 keer per dag in de ochtend in te nemen met een beetje water (elke dag rond dezelfde tijd tussen 7 en 9 uur in de ochtend). Het maakt niet uit of u de studie medicatie inneemt voor of na het eten.

*Stap 3: onderzoeken en metingen*

Voor het onderzoek kan van u gevraagd worden om een aantal keer extra naar uw ziekenhuis of dialysecentrum te komen. Dit is afhankelijk van uw situatie. De eerste 3 bezoeken en een eindbezoek kunnen plaatsvinden naast uw normale ziekenhuisbezoeken.

Bezoek 1 is om uw geschiktheid te bepalen en uw medische gegevens te verzamelen (geschiktheidsbezoek).

Bezoek 2 is om te loten in welke groep u gaat deelnemen (randomisatiebezoek)

Bezoek 3 is als extra veiligheidscontrole (veiligheidsbezoek).

Als u geschikt bent bevonden om mee te doen aan het onderzoek, zal er een randomisatiebezoek plaatsvinden. Tijdens dit tweede bezoek wordt door loting bepaald aan welke onderzoeksgroep u gaat deelnemen. Daarna krijgt u de studiemedicatie mee. Nadat u bent gestart met de studie medicatie, komt u na 2/3 weken (een veiligheidsbezoek), na 12 weken, na 26 weken en elke 6 maanden daarna voor een studiebezoek. Vanaf het bezoek na 12 weken zullen de bezoeken zoveel mogelijk gecombineerd worden met uw standaard ziekenhuisbezoeken. Een studie bezoek duurt ongeveer een uur.

Bloedafname:

Bij elk bezoek zal er bloed bij u worden afgenomen voor routine bloedonderzoek. Dat is ongeveer 20 ml per keer. Bij start van de behandeling (tijdens het randomisatie bezoek), na 12 weken en bij het eindbezoek wordt aanvullend nog 20 mL bloed afgenomen voor opslag van bloed, waarin later extra bepalingen gedaan kunnen worden.

Urine verzamelen:

Bij het geschikheidsbezoek, bij start van de behandeling (randomisatiebezoek), na 26 weken en elke 6 maanden daarna wordt u gevraagd om ochtend urine te verzamelen. De urineverzameling vindt plaats in de ochtend van het studiebezoek. Alleen voor dialyse patiënten geldt dat ze bij start van de behandeling (randomisatiebezoek) en elke 6 maanden daarna gedurende 24 uur urine moeten verzamelen (in plaats van ochtend urines) voor beoordelen van de rest nierfunctie en dialyse efficiëntie.

Bij het geschiktheidsbezoek doet de onderzoeker of uw arts een routinematig lichamelijk onderzoek (zoals bloeddruk, hartslag en gewicht) om uw gezondheid te controleren. Bij vrouwen die zwanger kunnen worden wordt een zwangerschapstest gedaan. U wordt elk bezoek gevraagd naar alle medicijnen die u gebruikt.

U wordt ook elk bezoek gevraagd naar mogelijke gebeurtenissen en bijwerkingen van de studie medicatie. Er wordt nagegaan of u bent gestart met dialyse en/of u opgenomen bent geweest in een ziekenhuis vanwege hartfalen. Bijwerkingen waar naar wordt gevraagd zijn blaasontstekingen, ontstekingen van geslachtsorganen en te lage bloedsuiker.

Wij vragen u om bij de start van de behandeling (randomisatie), na 6 maanden, na 1 jaar en daarna 1x per jaar twee vragenlijsten in te vullen. Eén vragenlijst bestaat uit 5 korte vragen en de andere vragenlijst bestaat uit 12 korte vragen. De vragen gaan over uw kwaliteit van leven. Het kost u ongeveer 10 minuten om deze vragenlijst in te vullen. De vragenlijsten kunnen digitaal ingevuld worden. Hiervoor vragen we uw toestemming om uw e-mailadres te gebruiken voor het verzenden van de vragenlijsten. Uw e-mailadres is zichtbaar voor het lokale onderzoeksteam en bij de opdrachtgever, het UMCG. U kunt eventueel de vragenlijsten ook op papier invullen.

Daarnaast vragen we u om bij de start van de behandeling (randomisatie), na 6 maanden, daarna na elke 6 maanden om een cognitieve test, de Symbol Digit Modalities Test, uit te voeren via een app op uw smartphone of op een tablet bij uw deelnemend ziekenhuis/dialysecentrum. Tijdens deze test krijgt u een symbool te zien en wordt u gevraagd het juiste cijfer aan te tikken op het scherm. U wordt gevraagd om in 90 seconden zo veel mogelijk symbolen en cijfers te koppelen.

Voor de test wordt gebruik gemaakt van een app ‘Orikami’. U wordt gevraagd de app te downloaden op uw eigen smartphone of u kunt de test op een tablet bij uw ziekenhuis of dialysecentrum maken. Bij het downloaden van de app wordt u gevraagd om akkoord te gaan met de algemene voorwaarden van de app. Daarnaast vragen we uw toestemming om uw e-mailadres te delen met Orikami zodat u de test kunt maken. Orikami beschermt uw gegevens in overeenstemming met de privacyregels in de Europese Unie. Uw e-mailadres is ook zichtbaar voor de opdrachtgever, het UMCG. Deelname aan deze extra test is niet verplicht. Mocht u hier geen toestemming voor geven, kunt u nog wel deelnemen aan de andere testen en onderzoeken.

**Bijlage C** laat zien wanneer welke metingen worden uitgevoerd.

*Wat is er anders dan bij gewone zorg?*

Het medicijn dat u tijdens dit onderzoek krijgt en de metingen die we doen, zijn puur in het kader van dit onderzoek, ze staan los van eventuele behandelingen en controles die u afspreekt met u huisarts of arts in het ziekenhuis.

Voorbeelden van verschillen met uw gewone zorg kunnen zijn dat:

• U voor extra bezoeken naar uw ziekenhuis of dialyse centrum komt

• U medicatie gaat slikken die u nu niet gebruikt (en u niet weet of het een echt of een nep medicijn is)

• Er elk bezoek extra bloed wordt afgenomen

• U elk bezoek urine inlevert

• U vragenlijsten invult

• U elk bezoek gevraagd wordt naar bijzondere gebeurtenissen (zoals ziekenhuisopname) en mogelijke bijwerkingen van de studiemedicatie

*Mogelijke ziekenhuisopname, chirurgische ingreep of een scan met contactvloeistof*

Tijdens deelname aan het onderzoek kan het zijn dat u een ziekenhuis wordt opgenomen, een chirurgische ingreep/operatie nodig heeft, of een scan krijgt met intraveneus contrast (bijv. CT-scan). We vragen uw behandeld arts dan contact op te nemen met de lokale onderzoeker aangezien het nodig zal zijn om tijdelijk te stoppen met het gebruik van uw studiemedicatie minimaal drie dagen voor uw opname in ziekenhuis of scan en/of volgens de richtlijnen van uw huisarts/medisch specialist. Deze informatie staat ook op de deelnemerskaart. Laat dit dan zo snel mogelijk weten aan uw huisarts/medisch specialist en onderzoekscoördinator. Uw studiemedicatie kan drie dagen na uw ingreep/scan weer worden gestart of zodra u zich beter voelt en normaal eet en drinkt en/of volgens de richtlijnen van uw huisarts/medisch specialist.

Als u zich niet goed voelt en naar een zorginstelling gaat, bijv. de huisartsenpraktijk of de Spoedeisende Hulp van een ziekenhuis, laat hen dan uw deelnemerskaart zien, aangezien dit zal verklaren dat u deelneemt aan een klinische studie en mogelijk dapagliflozine of placebo gebruikt.

**5. Welke afspraken maken we met u?**

We willen graag dat het onderzoek goed verloopt. Daarom willen graag de volgende afspraken met u maken:

- - U neemt het medicijn op de manier die de onderzoeker u heeft uitgelegd.
  - U doet tijdens dit onderzoek niet ook nog mee aan een ander medisch-wetenschappelijk onderzoek, tenzij u daarvoor toestemming heeft van uw studie arts. Dit is om u te beschermen tegen mogelijke schade als gevolg van bijvoorbeeld teveel bloed afnames, mogelijke medicatie-interacties of andere gevaren.
  - U komt naar iedere afspraak.
  - U draagt de deelnemerskaart van het onderzoek bij u. Bijvoorbeeld in uw portemonnee. Hierop staat dat u meedoet aan dit onderzoek. En wie men moet waarschuwen bij een noodsituatie. Laat deze kaart zien als u bij een (andere) arts komt.
  - U moet eraan denken om uw ongebruikte studie medicatie en alle lege potjes mee te nemen naar elk studiebezoek en te vertellen of er studie medicatie verloren of verdwenen is.
  - U neemt contact op met de onderzoeker in de volgende situaties:
  - U wilt andere medicijnen gaan gebruiken. Ook als dit homeopathische middelen, natuurgeneesmiddelen, vitaminen of geneesmiddelen van de drogist zijn.
  - U wordt in een ziekenhuis opgenomen of behandeld.
  - U krijgt plotseling problemen met uw gezondheid.
  - U wilt niet meer meedoen met het onderzoek.
  - Uw telefoonnummer, adres of e-mailadres verandert.

*Mag u of uw partner zwanger worden tijdens het onderzoek?*

Vrouwen die zwanger zijn of borstvoeding geven, kunnen niet meedoen aan dit onderzoek. Vrouwen mogen ook niet zwanger worden tijdens het onderzoek tot 4 weken na de laatste inname van de studiemedicatie. Er is namelijk geen onderzoek gedaan met dapagliflozine bij zwangere vrouwen of vrouwen die borstvoeding geven. Het is dus niet bekend of dapagliflozine veilig is voor ongeboren baby's en zuigelingen.

Als u vrouw bent, vraagt de onderzoeker naar de laatste keer dat u ongesteld was, om te onderzoeken of u in de menopauze bent. Als u niet in de menopauze bent, dan voeren we een zwangerschapstest uit. Hiervoor moet u op een teststrookje plassen.

Aanvaardbare methoden voor anticonceptie omvatten consistent en correct gebruik van:

• Orale anticonceptie (‘de pil’)

• Implanteerbaar of injecteerbaar anticonceptiemiddel

• Intra-uterien device/intra-uterien systeem (IUD's/IUS's)

• Transdermale pleister

• Dubbele barrièremethode (d.w.z. twee methoden die tegelijkertijd worden gebruikt, zoals een mannencondoom in combinatie met een vrouwelijk diafragma / cervicale kap plus zaaddodend schuim/gel/film/crème/zetpil)

Mannelijke deelnemers dienen af te zien van geslachtsgemeenschap met een vrouwelijke partner zonder bescherming vanaf het begin van de eerste behandelingsperiode tot ten minste 3 maanden na de laatste inname van de onderzoeksmedicatie. Voor mannelijke deelnemers van wie de partner al zwanger is, moet de man een condoom gebruiken tijdens de duur van dit onderzoek en gedurende 3 maanden daarna. Mannelijke deelnemers mogen geen sperma doneren tijdens de duur van dit onderzoek en gedurende ten minste 3 maanden na het follow-upbezoek van dit onderzoek.

*Toch zwanger?*

Wordt u toch zwanger tijdens het onderzoek? Laat dit dan meteen weten aan de onderzoeker. U moet dan in overleg met de onderzoeker zo snel mogelijk stoppen met dit onderzoek.

**6. Van welke bijwerkingen, nadelige effecten of ongemakken kunt u last krijgen?**

U kunt last hebben van de handelingen tijdens het onderzoek. Bijvoorbeeld bloedafname kan pijnlijk zijn of u kunt een blauwe plek krijgen. We zullen daarom bloedafnames zoveel mogelijk combineren met bloedafnames die in het kader van normale patiëntenzorg plaatsvinden.

Dapagliflozine kan bijwerkingen geven. Het kan dat u alle, enkele of geen van de bijwerkingen krijgt die hieronder staan aangegeven. Deze bijwerkingen zijn bekend vanuit andere patiëntengroepen. We weten nog niet of dit hetzelfde is voor u.

De volgende bijwerkingen zijn bekend (u leest hierover meer in **bijlage D**):

- - Toename van de hoeveelheid urine en/of dorstgevoel
  - Blaasontsteking
  - Infecties van de vagina of penis en/of urinebuis

Dapagliflozine kan ook bijwerkingen hebben die we nu nog niet weten. Het gebruik van een onderzoeksmiddel brengt altijd risico’s met zich mee. Daarvoor wordt uw gezondheid goed in de gaten gehouden. Vertel alle bijwerkingen aan de onderzoeker, zodat u eventueel passende zorg kunt ontvangen. Het melden van de bijwerkingen is belangrijk voor uw eigen bescherming en voor het voorkomen van problemen. Meer informatie over dapagliflozine staat in de bijsluiter. Doet u mee aan het onderzoek? Dan krijgt u de bijsluiter mee bij het middel. Aangezien dapagliflozine tabletten lactose bevatten kunt u hier ongemak van ondervinden als u lactose-intolerantie heeft.

**7. Wat zijn de voordelen en de nadelen als u meedoet aan het onderzoek?**

Meedoen aan het onderzoek kan voordelen en nadelen hebben. Hieronder zetten we ze op een rij. Denk hier goed over na, en praat erover met anderen.

Als u aan dit onderzoek meedoet, wil dat niet zeggen dat uw ziekte zal genezen of dat u minder last heeft van uw ziekte. Maar als u meedoet, helpt u de onderzoekers om meer inzicht te krijgen in de behandeling van hart- en nieraandoeningen. Dapagliflozine kan verschillende positieve effecten geven, namelijk het verlagen van de bloeddruk, het verlagen van de hoeveelheid eiwit in de urine, het voorkomen van nierfalen, hartfalen en sterfte, maar zeker is dat niet.

Meedoen aan het onderzoek kan nadelen hebben:

- - U kunt last krijgen van bijwerkingen of negatieve effecten van dapagliflozine, zoals hierboven beschreven.
  - Meedoen aan het onderzoek kost u extra tijd.
  - U moet zich houden aan de afspraken die horen bij het onderzoek.

Het is mogelijk dat er tijdens het onderzoek toevallig iets wordt ontdekt dat niet direct van belang is voor het onderzoek maar wel voor uw gezondheid. In dit geval zal uw eigen huisarts of specialist met u bespreken wat er verder moet gebeuren. De kosten hiervan vallen onder uw eigen zorgverzekering.

*Wilt u niet meedoen?*

U beslist zelf of u meedoet aan het onderzoek. Deelname is vrijwillig. Wilt u niet meedoen? Dan krijgt u de gewone behandeling voor uw aandoening zoals u die nu ook krijgt.

**8. Wanneer stopt het onderzoek?**

De onderzoeker laat het u weten als er nieuwe informatie over het onderzoek komt die belangrijk voor u is. De onderzoeker vraagt u daarna of u blijft meedoen.

In deze situaties stopt voor u het onderzoek:

- - Het einde van het hele onderzoek is bereikt.
  - U bent zwanger geworden.
  - U wilt zelf stoppen met het onderzoek. Dat mag op ieder moment. Meld dit dan meteen bij de onderzoeker. U hoeft er niet bij te vertellen waarom u stopt. De onderzoeker zal u nog wel uitnodigen voor een eindbezoek.
  - De onderzoeker vindt het beter voor u om te stoppen. De onderzoeker zal u nog wel uitnodigen voor een eindbezoek.
  - Een van de volgende instanties besluit dat het onderzoek moet stoppen:
  - UMCG
  - de overheid, of
  - de medisch-ethische commissie die het onderzoek beoordeelt.

*Wat gebeurt er als u stopt met het onderzoek?*

De onderzoekers gebruiken de gegevens en het lichaamsmateriaal (bloed en urine) die tot het moment van stoppen zijn verzameld. Als u wilt, kan verzameld lichaamsmateriaal worden vernietigd. Geef dit door aan de onderzoeker.

**9. Wat gebeurt er na het onderzoek?**

*Kunt u de medicijnen blijven gebruiken?*

Indien u dat wilt, dan kunt u de medicijnen die u heeft gebruikt bij het onderzoek, na het onderzoek blijven doorgebruiken. Dat zal dan gewoon op recept gebeuren.

*Krijgt u de resultaten van het onderzoek?*

Ongeveer 12 maanden na het stoppen van het onderzoek laat de onderzoeker u weten wat de belangrijkste uitkomsten zijn van het onderzoek. De onderzoeker zal u dan ook vertellen in welke groep u zat. Wilt u dit niet weten? Zeg dat dan tegen de onderzoeker. Hij zal het u dan niet vertellen.

**10. Wat doen we met uw gegevens** **en lichaamsmateriaal?**

Doet u mee met het onderzoek? Dan geeft u ook toestemming om uw gegevens en lichaamsmateriaal te verzamelen, gebruiken en bewaren. Uw deelname aan dit onderzoek wordt ook in uw ziekenhuis of dialysecentrum in uw medisch dossier vastgelegd. Dit is voor veiligheidsdoeleinden.

*Welke gegevens bewaren we?*

We bewaren deze gegevens:

- uw naam

- uw geslacht

- uw adres

- uw geboortedatum

- gegevens over uw gezondheid

- (medische) gegevens die we tijdens het onderzoek verzamelen

*Welk lichaamsmateriaal bewaren we?*

We bewaren kleine hoeveelheden bloed en ochtendurine.

*Waarom verzamelen, gebruiken en bewaren we uw gegevens en lichaamsmateriaal?*

We verzamelen, gebruiken en bewaren uw gegevens en uw lichaamsmateriaal om de vragen van dit onderzoek te kunnen beantwoorden. Wij vragen voor het gebruik van uw gegevens en lichaamsmateriaal uw toestemming. Indien u dat niet wilt, kunt u niet deelnemen aan dit onderzoek.

*Hoe beschermen we uw privacy?*

Om uw privacy te beschermen geven wij uw gegevens en uw lichaamsmateriaal een code. Op al uw gegevens en lichaamsmateriaal zetten we alleen deze code. De sleutel van de code bewaren we op een beveiligde plek in het UMC Groningen. Als we uw gegevens en lichaamsmateriaal verwerken, gebruiken we steeds alleen die code. Ook in rapporten en publicaties over het onderzoek kan niemand terughalen dat het over u ging.

*Wie kunnen uw gegevens zien?*

Sommige personen kunnen wel uw naam en andere persoonlijke gegevens zonder code inzien. Dit zijn mensen die controleren of de onderzoekers het onderzoek goed en betrouwbaar uitvoeren. Deze personen kunnen bij uw gegevens komen:

- - Leden van de commissie die de veiligheid van het onderzoek in de gaten houdt.
  - Een controleur die voor de opdrachtgever werkt.
  - Nationale en internationale toezichthoudende autoriteiten. Bijvoorbeeld de Inspectie Gezondheidszorg en Jeugd en het Europese Medicijnagentschap (EMA).

Deze personen houden uw gegevens geheim. Wij vragen u voor deze inzage toestemming te geven.

*Hoelang bewaren we uw gegevens en lichaamsmateriaal?*

We bewaren uw gegevens 25 jaar in het UMC Groningen. Uw lichaamsmateriaal bewaren we ook in het UMC Groningen. Het wordt 25 jaar bewaard om daarop in de loop van dit onderzoek nog nieuwe bepalingen te kunnen doen die te maken hebben met dit onderzoek. Zodra dit niet meer nodig is, vernietigen we uw lichaamsmateriaal.

*Mogen we uw gegevens en lichaamsmateriaal gebruiken voor ander onderzoek?*

Uw gegevens en uw (overgebleven) lichaamsmateriaal kunnen na afloop van dit onderzoek ook nog van belang zijn voor ander wetenschappelijk onderzoek op het gebied van nierfalen en hartfalen. Daarvoor zullen uw gegevens en lichaamsmateriaal 25 jaar worden bewaard. In het toestemmingformulier geeft u aan of u dit goed vindt.

*Wat gebeurt er bij onverwachte ontdekkingen?*

Tijdens het onderzoek kunnen we toevallig iets vinden dat belangrijk is voor uw gezondheid. De onderzoeker neemt dan contact op met uw huisarts en/of behandelend specialist. U bespreekt dan met uw huisarts of specialist wat er moet gebeuren. U geeft met het formulier toestemming voor het informeren van uw huisarts of specialist.

*Kunt u uw toestemming voor het gebruik van uw gegevens weer intrekken?*

U kunt uw toestemming voor het gebruik van uw gegevens op ieder moment intrekken. Dit geldt voor het gebruik in dit onderzoek en voor het gebruik in ander onderzoek. Maar let op: trekt u uw toestemming in, en hebben onderzoekers dan al gegevens verzameld voor een onderzoek? Dan mogen zij deze gegevens nog wel gebruiken. Voor uw lichaamsmateriaal geldt dat de onderzoekers dit vernietigen nadat u uw toestemming intrekt. Maar zijn er dan al metingen gedaan met uw lichaamsmateriaal? Dan mag de onderzoeker de resultaten daarvan blijven gebruiken.

*We sturen uw gegevens en lichaamsmateriaal naar landen buiten de Europese Unie*

Tijdens en na afloop van dit onderzoek kunnen wij uw gecodeerde gegevens en lichaamsmateriaal ook naar landen buiten de Europese Unie opsturen voor opslag van de gegevens, en analyse van bloed en urine om een beter inzicht te krijgen in de werking van Dapagliflozine. In die landen gelden niet de privacyregels van de Europese Unie, maar uw privacy zal op een gelijkwaardig niveau worden beschermd.

*Wilt u meer weten over uw privacy?*

- - Wilt u meer weten over uw rechten bij de verwerking van persoonsgegevens? Kijk dan op [www.autoriteitpersoonsgegevens.nl](http://www.autoriteitpersoonsgegevens.nl).
  - Heeft u vragen over uw rechten? Of heeft u een klacht over de verwerking van uw persoonsgegevens? Neem dan contact op met degene die verantwoordelijk is voor de verwerking van uw persoonsgegevens. Voor uw onderzoek is dat:
  - De functionaris gegevensbescherming in het UMC Groningen, te bereiken via [privacy@umcg.nl](mailto:privacy@umcg.nl)
  - Als u klachten heeft over de verwerking van uw persoonsgegevens, raden we u aan om deze eerst te bespreken met het onderzoeksteam. U kunt ook naar de Klachtenfunctionaris van het UMCG gaan. Of u dient een klacht in bij de Autoriteit Persoonsgegevens.

**11. Krijgt u een vergoeding als u meedoet aan het onderzoek?**

De onderzoeksmiddelen, extra testen en behandeling voor het onderzoek kosten u niets. U krijgt ook geen vergoeding als u meedoet aan dit onderzoek. Wel krijgt u een vergoeding voor uw reiskosten indien u voor extra bezoeken naar uw ziekenhuis/dialyse centrum komt.

**12. Bent u verzekerd tijdens het onderzoek?**

Voor iedereen die meedoet aan dit onderzoek is een verzekering afgesloten. Dit is de standaard verzekering van het UMC Groningen die betaalt voor schade door het onderzoek. Maar niet voor alle schade. In **bijlage B** vindt u meer informatie over de verzekering en de uitzonderingen. Daar staat ook aan wie u schade kunt melden.

**13. We informeren uw huisarts en/of behandelend specialist**

De onderzoeker stuurt uw huisarts en/of behandelend medisch specialist een brief/e-mail om te laten weten dat u meedoet aan het onderzoek. Dit is voor uw eigen veiligheid. Uw huisarts en eventueel behandelend medisch specialist worden geïnformeerd over eventuele toevalsbevindingen. Als u dit niet goed vindt, kunt u niet meedoen aan dit onderzoek.

**14. Heeft u vragen?**

Vragen over het onderzoek kunt u stellen aan de onderzoeker en het onderzoeksteam. Wilt u advies van iemand die er niet bij betrokken is? Neem dan contact op met dr. P. van Dijk (diabetes arts in het UMC Groningen). Hij weet veel over het onderzoek, maar werkt niet mee aan dit onderzoek.

Heeft u een klacht? Bespreek dit dan met de onderzoeker of de arts die u behandelt. Wilt u dit liever niet? Ga dan naar de klachtenfunctionaris/klachtencommissie van het UMCG. In **bijlage A** staat waar u die kunt vinden.

**15. Hoe geeft u toestemming voor het onderzoek?**

U kunt eerst rustig nadenken over dit onderzoek. Daarna vertelt u de onderzoeker of u de informatie begrijpt en of u wel of niet wilt meedoen. Wilt u meedoen? Dan vult u het toestemmingsformulier in dat u bij deze informatiebrief vindt. U en de onderzoeker krijgen allebei een getekende versie van deze toestemmingsverklaring.

**16. Bijlagen bij deze informatie**

A. Contactgegevens UMC Groningen

B. Informatie over de verzekering

C. Schema onderzoekshandelingen

D. Bijwerkingen/risico’s

E. Toestemmingsformulier

**Bijlage A: contactgegevens voor Universitair Medisch Centrum Groningen**

**Contactgegevens onderzoekers:**

Prof. Dr. R.T. Gansevoort Prof. Dr. H.J. Lambers Heerspink

Internist-nefroloog Klinisch farmacoloog

Telefoon: 050-3616161 Telefoon: 050-3616161

E-mail: r.t.gansevoort@umcg.nl E-mail: [h.j.lambers.heerspink@umcg.nl](mailto:h.j.lambers.heerspink@umcg.nl)

Universitair Medisch Centrum Groningen Universitair Medisch Centrum Groningen

Interne geneeskunde, afdeling nefrologie Klinische farmacie en farmacologie

Hanzeplein 1 Hanzeplein 1

9713 GZ, Groningen 9713 GZ, Groningen

**Onafhankelijk arts:**

Dr. P. van Dijk

Internist-endocrinoloog

Telefoon: 050-3616161

E-mail: [p.r.van.dijk@umcg.nl](mailto:p.r.van.dijk@umcg.nl)

Universitair Medisch Centrum Groningen

Interne geneeskunde, afdeling endocrinologie

Hanzeplein 1

9713 GZ, Groningen

Klachten: <https://www.umcg.nl/-/contact-met-het-umcg/vertel-het-ons/klachten>

Functionaris voor de Gegevensbescherming van het UMCG: Piet Dinjens en Boudien Sieperda, te bereiken via [privacy@umcg.nl](mailto:privacy@umcg.nl)

Voor meer informatie over uw rechten: <https://www.umcg.nl/-/rechten-regels-patient> en aanvullende informatie omtrent uw privacy: <https://www.umcg.nl/-/privacystatement-umcg>

**Bijlage B: informatie over de verzekering**

Het UMCG heeft een verzekering afgesloten voor iedereen die meedoet aan onderzoek. Deze verzekering betaalt schade als die door het onderzoek is veroorzaakt. Het gaat om schade die ontstaat tijdens het onderzoek, of binnen 4 jaar na het onderzoek. U moet schade binnen 4 jaar melden bij de verzekeraar.

Heeft u schade door het onderzoek? Meld dit dan direct (telefonisch/mail/post) met de verzekeraar, of u kunt zich wenden tot een contactpersoon van het UMCG.

De verzekeraar van het onderzoek is:

Naam: Centramed

Adres: Maria Montessorilaan 9, 2719 DB Zoetermeer

Postadres: Postbus 7374, 2701 AJ Zoetermeer

Telefoonnummer: 070-3017070

E-mail: info@centramed.nl

Polisnummer: 624.529.102

De verzekering betaalt maximaal €650.000 per persoon en €5.000.000 voor het hele onderzoek en €7.500.000 per jaar voor alle onderzoeken van dezelfde opdrachtgever.

Let op: de verzekering dekt de volgende schade **niet**:

- - Schade door een risico waarover we u informatie hebben gegeven in deze brief. Maar dit geldt niet als het risico groter bleek te zijn dan we van tevoren dachten. Of als het risico heel onwaarschijnlijk was.
  - Schade aan uw gezondheid die ook zou zijn ontstaan als u niet aan het onderzoek had meegedaan.
  - Schade die ontstaat doordat u aanwijzingen of instructies niet of niet goed opvolgde.
  - Schade aan de gezondheid van uw kinderen of kleinkinderen.
  - Schade door een behandelmethode die al bestaat. Of door onderzoek naar een behandelmethode die al bestaat.

Deze bepalingen staan in het 'Besluit verplichte verzekering bij medisch-wetenschappelijk onderzoek met mensen 2015'. Dit besluit staat in de Wettenbank van de overheid (<https://wetten.overheid.nl>).

**Bijlage C: Schema onderzoekshandelingen**

|  | | *Screening* |  | *Behandelingsperiode* | | | | | | *(Vervroegde) Beëindiging* |
| --- | --- | --- | --- | --- | --- | --- | --- | --- | --- | --- |
| **Bezoeken** | | **1**  **Geschiktheids-bezoek** | **2**  **Randomisatie-bezoek** | | **3**  **veiligheidsbezoek** | **4** | **5** | **6** | **7, 8, 9, 10, 11, 12 …** | **eindbezoek** |
| ***Week*** | | *-2* | *0* | | *2* | *12* | *6 maanden* | *1 jaar* | *Elke 6 maanden* |  |
| Geïnformeerde toestemming | | x |  | |  |  |  |  |  |  |
| Randomisatie | |  | x | |  |  |  |  |  |  |
| Vragenlijsten | |  | x | |  |  | x | x | x* | x |
| Cognitieve test via app Orikami | |  | x | |  |  | x | x | x* | x |
| Medische voorgeschiedenis | | x |  | |  |  |  |  |  | x |
| Medicatie gebruik navragen | | x | x | | x | x | x | x | x | x |
| Vitale functies meten | | x | x | | x | x | x | x | x | x |
| Lichamelijk onderzoek | | x | x** | | x** | x** | x** | x** | x** | x** |
| Zwangerschapstest | | x | x** | | x** | x** | x** | x** | x** | x** |
| Bloedafname | | x | x | | x | x | x | x | x | x |
| Ochtendurine monsters | | x | x | |  | x | x | x | x | x |
| 24uurs urine verzamelen  *alleen voor dialyse patiënten* | |  | x | |  | x | x | x | x | x |
| Opslag bloed en urine | |  | x | |  | x |  |  |  | x |
| Uitgifte onderzoeksmedicatie | |  | x | | x | x | x | x | x |  |
| Controle onderzoeksmedicatie | |  |  | | x | x | x | x | x | x |
| Bijwerkingen controleren | |  | x | | x | x | x | x | x | x |
| Navragen gebeurtenissen | |  |  | | x | x | x | x | x | x |
|  | * één keer per jaar  ** alleen wanneer noodzakelijk | | | | | | | | | |

**Bijlage D: Bijwerkingen, nadelige effecten en ongemakken**

Dapagliflozine (Forxiga^®^) wordt momenteel wereldwijd voorgeschreven voor de behandeling van diabetes, hart- en vaatziekten en nierziekten. Bijwerkingen, risico’s en eventuele nadelen zijn bekend en zorgvuldig gedocumenteerd. Dapagliflozine is nog niet bestudeerd bij mensen met een ernstig gestoorde nierfunctie (lager dan 30 ml/min/1.73m2, dialyse patiënten en transplantatie patiënten). Het kan zijn dat u last krijgt van geen, enkele of alle bijwerkingen die hieronder worden genoemd.

*De volgende bijwerkingen komen vaak (1-10%) voor:*

- **Verhoging van de hoeveelheid urine**:

Dapagliflozine zorgt ervoor dat suiker in de urine terechtkomt, wat zorgt voor meer aanmaak van urine. Symptomen: u moet vaker plassen en krijgt dorst. Tekenen van verlies van te veel lichaamsvocht zijn een lage bloeddruk en duizeligheid.

- **Blaasontsteking:**

Dapagliflozine kan het risico op ontstekingen van de blaas vergroten.

Symptomen: ongemak met plassen, verhoogde aandrang om te plassen, vaker plassen of koorts.

- **Infecties van de geslachtsorganen:**

Dapagliflozine kan het risico op schimmelinfecties van de vagina, vulva of penis vergroten. Deze infecties komen vaker voor bij vrouwen en bij mensen met een voorgeschiedenis van deze infecties.

Symptomen: pijn of een jeukend gevoel, een afscheiding of uitslag in de schaamstreek.

*De volgende bijwerkingen komen weinig voor:*

- **Huiduitslag en/of jeuk, overgevoeligheid**
- **Een lage bloedsuikerspiegel (hypoglykemie) is waarschijnlijk zeer zeldzaam:**

Deze bijwerking komt alleen in uitzonderlijke gevallen voor bij patiënten met suikerziekte.

Symptomen: overmatige zweten, hartkloppingen, beven, duizeligheid, wazig zien, malaise (zoals vermoeidheid, zwakte) en lage bloedsuikerspiegel.

- **Verzuring van het bloed (diabetische ketoacidose) is waarschijnlijk zeer zeldzaam:**

Dapagliflozine kan het risico op verzuring van het bloed veroorzaken. Deze bijwerking komt alleen in uitzonderlijke gevallen voor bij patiënten met suikerziekte type 2. Mocht u last krijgen van onderstaande symptomen, dan moet u direct contact met ons opnemen.

Symptomen: misselijkheid, overgeven, buikpijn, malaise (zoals vermoeidheid, zwakte), kortademigheid en hoge bloedsuikerspiegel.

**Bijlage E: Toestemmingsformulier proefpersoon**

Behorende bij **de RENAL LIFECYCLE studie**: Een placebo gecontroleerde studie om het effect van dapagliflozine op nier- en hartfalen te onderzoeken bij nierpatiënten

- Ik heb de informatiebrief gelezen. Ook kon ik vragen stellen. Mijn vragen zijn goed genoeg beantwoord. Ik had genoeg tijd om te beslissen of ik meedoe.
- Ik weet dat meedoen vrijwillig is. Ook weet ik dat ik op ieder moment kan beslissen om toch niet mee te doen met het onderzoek. Of om ermee te stoppen. Ik hoef dan niet te zeggen waarom ik wil stoppen.
- Ik geef de onderzoeker toestemming om mijn huisarts en/of specialist(en) die mij behandelt te laten weten dat ik meedoe aan dit onderzoek.
- Ik geef de onderzoeker toestemming om informatie op te vragen bij mijn huisarts en/of specialist(en) die mij behandelt over mijn medicijngebruik en behandelmethodes zolang het te maken heeft met de veiligheid en/of algemene vraagstelling van het onderzoek.
- Ik geef de onderzoeker toestemming om mijn huisarts of specialist informatie te geven over onverwachte bevindingen uit het onderzoek die van belang zijn voor mijn gezondheid.
- Ik geef de onderzoekers toestemming om mijn gegevens en lichaamsmateriaal te verzamelen, gebruiken en eventueel te delen met andere onderzoekers, zowel nationaal als internationaal, ook buiten Europa. De onderzoekers doen dit alleen om de onderzoeksvragen die te maken hebben met het onderzoek te beantwoorden. Het delen van data en lichaamsmateriaal gebeurt alleen gecodeerd.
- Ik weet dat voor de controle van het onderzoek sommige mensen al mijn gegevens kunnen inzien. Die mensen staan in deze informatiebrief. Ik geef deze mensen toestemming om mijn gegevens in te zien voor deze controle.
- Ik weet dat ik niet zwanger mag worden tijdens het onderzoek en tot 4 weken na de laatste inname van de studiemedicatie en de onderzoeker heeft besproken hoe ik het beste voorkom dat ik zwanger word.
- Ik wil meedoen aan dit onderzoek.

Wilt u in de tabel hieronder ja of nee aankruisen?

| Ik geef toestemming om mijn gegevens te bewaren om dit te gebruiken voor ander onderzoek, zoals in de informatiebrief staat. | Ja ☐ | Nee ☐ |
| --- | --- | --- |
| Ik geef toestemming om mijn (overgebleven) lichaamsmateriaal te bewaren om  dit te gebruiken voor ander onderzoek, zoals in de informatiebrief staat. | Ja ☐ | Nee ☐ |
| Ik geef toestemming om mijn data te koppelen met registraties van ziekenhuisopnames, ziekte, behandeling en sterfte.  Bijvoorbeeld:  VEKTIS (nationale registratie van ziekenhuisopname diagnoses), RENINE (nationale registratie  van dialyse patiënten), NOTR (nationale registratie van patiënten met een getransplanteerd  orgaan), CBS (nationale registratie van overlijdensoorzaken) mits het in verband is met de  algemene vraagstelling van het onderzoek. | Ja ☐ | Nee ☐ |
| Ik geef toestemming om mij eventueel na dit onderzoek te vragen of ik wil meedoen  met een vervolgonderzoek. | Ja ☐ | Nee ☐ |
| Ik geef de onderzoekers toestemming om na het onderzoek te laten weten in welke  groep ik zat wanneer deze informatie bekend is. | Ja ☐ | Nee ☐ |
| Ik geef toestemming om mijn e-mailadres te gebruiken voor het digitaal invullen  van de vragenlijsten.  e-mailadres: ___________________________________________________ | Ja ☐ | Nee ☐ |
| Ik geef toestemming om deel te nemen aan het extra onderzoek met de cognitieve  test en mijn e-mailadres mag daarvoor gebruikt worden.  e-mailadres: ___________________________________________________ | Ja ☐ | Nee ☐ |

Mijn naam is (proefpersoon): …………………………………………….…………………….……………………Handtekening: Datum : __ / __ / __

­­­­­­­_____________________________________________________________________________________

Ik verklaar dat ik deze proefpersoon volledig heb geïnformeerd over het genoemde onderzoek.

Wordt er tijdens het onderzoek informatie bekend die de toestemming van de proefpersoon kan beïnvloeden? Dan laat ik dit op tijd weten aan deze proefpersoon.

Naam onderzoeker (of diens vertegenwoordiger):……………………………….…………… ……………………

Handtekening: Datum: __ / __ / __

­­­­­­­­­_____________________________________________________________________________________

Aanvullende informatie is gegeven door:

Naam:…………………………… ………………….…………………….…………………….………………………

Functie:……………………….…………………….…………………….…………………….……………………….

Handtekening: Datum: __ / __ / __

­­­­­­­­­*De proefpersoon krijgt een informatiebrief mee en een getekende versie van het toestemmingsformulier.*

1. Hochwirksame Empfängnisverhütungsmethoden sind unter anderem Implantate, Injektionen, orale Kombinationspräparate zur Empfängnisverhütung (mit deren Einnahme mindestens 3 Monate vor Einschluss in die Studie begonnen wurde), Intrauterinpessare (häufig auch unter der Bezeichnung Spirale bekannt), sterilisierter Partner oder echte und absolute Enthaltsamkeit (d.h. keine Kalender- oder Temperaturmethode). [↑](#footnote-ref-1)
